# Elimination of visceral leishmaniasis in the Indian subcontinent: a comparison of predictions from three transmission models

**DOI:** 10.1016/j.epidem.2017.01.002

**Published:** 2017-03

**Authors:** Epke A. Le Rutte, Lloyd A.C. Chapman, Luc E. Coffeng, Sarah Jervis, Epco C. Hasker, Shweta Dwivedi, Morchan Karthick, Aritra Das, Tanmay Mahapatra, Indrajit Chaudhuri, Marleen C. Boelaert, Graham F. Medley, Sridhar Srikantiah, T. Deirdre Hollingsworth, Sake J. de Vlas

**Affiliations:** a Department of Public Health, Erasmus MC, University Medical Center Rotterdam, P.O. Box 2040, 3000 CA Rotterdam, The Netherlands; b School of Life Sciences, University of Warwick, Gibbet Hill Campus, Coventry CV4 7AL, United Kingdom; c Institute of Tropical Medicine, Nationalestraat 155, 2000 Antwerp, Belgium; d CARE India Solutions for Sustainable Development, Patna, Bihar, India; e London School of Hygiene and Tropical Medicine, Keppel Street, London WC1E 7HT, United Kingdom

**Keywords:** Visceral leishmaniasis, Kala-azar, Elimination, Mathematical modelling, Indoor residual spraying, Detection and treatment, Indian subcontinent, Neglected tropical disease, Sandfly, Transmission dynamics, Predictions

## Abstract

We present three transmission models of visceral leishmaniasis (VL) in the Indian subcontinent (ISC) with structural differences regarding the disease stage that provides the main contribution to transmission, including models with a prominent role of asymptomatic infection, and fit them to recent case data from 8 endemic districts in Bihar, India. Following a geographical cross-validation of the models, we compare their predictions for achieving the WHO VL elimination targets with ongoing treatment and vector control strategies. All the transmission models suggest that the WHO elimination target (<1 new VL case per 10,000 capita per year at sub-district level) is likely to be met in Bihar, India, before or close to 2020 in sub-districts with a pre-control incidence of 10 VL cases per 10,000 people per year or less, when current intervention levels (60% coverage of indoor residual spraying (IRS) of insecticide and a delay of 40 days from onset of symptoms to treatment (OT)) are maintained, given the accuracy and generalizability of the existing data regarding incidence and IRS coverage. In settings with a pre-control endemicity level of 5/10,000, increasing the effective IRS coverage from 60 to 80% is predicted to lead to elimination of VL 1–3 years earlier (depending on the particular model), and decreasing OT from 40 to 20 days to bring elimination forward by approximately 1 year. However, in all instances the models suggest that *L. donovani* transmission will continue after 2020 and thus that surveillance and control measures need to remain in place until the longer-term aim of breaking transmission is achieved.

## 1 Introduction

Visceral leishmaniasis (VL), also known as kala-azar, is the world’s deadliest parasitic disease after malaria (Mathers et al., 2007). From 2004–2008 there were an estimated 200,000–400,000 cases and 20,000–40,000 deaths per year globally (Alvar et al., 2012). Historically, most VL cases occur in the Indian subcontinent (ISC), where the causative parasite *Leishmania donovani* is transmitted by *Phlebotomus argentipes* sandflies and the disease is considered to infect humans only (Dinesh et al., 2009, Swaminath et al., 1942). However, since 2012, there has been a significant decline in the number of VL cases identified in the ISC, attributed usually to interventions and socio-economic improvements (Chowdhury et al., 2014, Anon., 2016, WHO, 2015, Sheets et al., 2010). The World Health Organization (WHO) has targeted VL for elimination as a public health problem in the ISC by 2020 (WHO, 2015). This is defined as <1 new VL case per 10,000 capita per year at sub-district (block) level. In the rest of the world, where VL is mainly zoonotic and caused by another parasite species, the WHO has not set any elimination target but aims for 100% detection and treatment of human cases. Current interventions in the ISC focus on reducing transmission through vector control, mainly by indoor residual spraying (IRS) of insecticides, and early detection and treatment of cases (World Health Organization, 2014). In 2012, the London Declaration on Neglected Tropical Diseases endorsed the WHO elimination target on VL and pledged to increase research, funding, supplies and awareness to combat this disease (Uniting to Combat Neglected Tropical Diseases, 2012). It has been estimated that the health and economic gains from reaching the WHO targets for VL will be enormous (de Vlas et al., 2016). The governments of India, Bangladesh, Nepal, Bhutan and Thailand have signed a memorandum of understanding setting an ambitious goal of eliminating VL as a public health problem (at sub-district level in India and Bangladesh, and district-level in Nepal and Bhutan) by or before the end of 2017 (World Health Organization South-East Asia, 2014). Incidence of VL in Bhutan and Thailand is currently very low and limited to sporadic cases. Nepal reached the targeted low incidence level in 2014 and has sustained it for 2 years (WHO, 2015). Even in Bangladesh and India the target-level incidence was reached in nearly 90% and 70% of endemic sub-districts respectively by 2015 (WHO, 2015). Nevertheless, to achieve the target in the remaining endemic sub-districts in India, special attention must be paid to the state of Bihar, which borders Nepal and accounts for 60–90% of cases in the ISC (World Health Organization, 2011) and about 80% of cases and 90% of deaths in India (Anon., 2016). Hence, in this study we focus on the VL elimination status and control strategies in Bihar.

Mathematical models capturing disease transmission dynamics and control measures have proven to be useful tools in predicting the feasibility of achieving elimination targets with existing strategies (Hollingsworth et al., 2015, Rock et al., 2015, Hirve et al., 2016, Rock et al., 2016). Deterministic VL transmission models have been developed previously based on the KalaNet dataset from Bihar (India) and Nepal (Stauch et al., 2011, Stauch et al., 2014). More recently, Le Rutte et al. (Le Rutte et al., 2016) published a set of 3 age-structured model variants, each with individuals from a different disease stage being the main contributors to transmission: asymptomatic individuals, previously immune individuals in whom infection has reactivated, and individuals with post-kala-azar dermal leishmaniasis (PKDL). A sensitivity analysis for the duration of immunity was included, as both the disease stage which contains the main contributors to transmission and the duration of immunity remain unknown factors in the transmission dynamics of VL (Hirve et al., 2016). Available data on the impact of IRS on VL incidence suggested that the most accurate predictions are given by the model variant in which asymptomatic individuals are the main contributors to transmission, with which elimination of VL (annual incidence rate of <1 per 10,000 per year) by 2017 was predicted to be feasible only in settings that experience optimal IRS (continuously implemented from 2012 onwards) and have a maximum baseline endemicity of 5–10 VL cases per 10,000 capita per year. In highly endemic settings (<20 VL cases per 10,000) and in settings with sub-optimal IRS that are facing challenges with IRS implementation, coverage and insecticide resistance, additional interventions will be required (Le Rutte et al., 2016). Chapman et al. (2015) have recently estimated key epidemiological parameters for VL, including the duration of asymptomatic infection and the proportion of asymptomatic individuals who develop clinical symptoms, by fitting a multi-state Markov model for the natural history of VL to serological and case data from a highly endemic region of Bangladesh (Bern et al., 2007). It was estimated that asymptomatic infection lasts 5 months on average and approximately 1 in 7 asymptomatic individuals progress to VL. However, the extent to which these parameters depend on geographical location, endemicity, and other risk factors remains unclear.

To improve the robustness of predictions of the impact of intervention strategies against VL it is vital to compare and combine the outcomes of different mathematical modelling approaches, as has been done previously for HIV (Hontelez et al., 2013, Eaton et al., 2012, Brisson et al., 2003). Here we present the first VL modelling comparison study in which we compare the predictions from (1) the VL transmission model variant of those developed by Le Rutte et al. (2016) that provides the most accurate predictions, (2) a similar model in which symptomatic individuals are the sole contributors to transmission, and (3) a newly developed transmission model based on the simplified model of the natural history of VL presented by Chapman et al. (2015). The three models were fitted to VL case data collected by CARE India in 2012 and 2013 from 8 endemic districts in Bihar, India (Das et al., 2016, Jervis et al., 2017), that are currently under intensive vector control with IRS. The models were compared via their predictive ability in a geographical cross-validation. The models were then used to predict whether the elimination target could be achieved in each district. We further predicted whether the elimination target could be achieved in settings with different pre-control endemicity levels, using current and improved interventions.

## 2 Methods

### 2.1 Mathematical models

The modelling study described in this paper was performed by two research groups: Erasmus MC, Department of Public Health in Rotterdam, The Netherlands and the Warwick Infectious Disease Epidemiology Research (WIDER) group, University of Warwick, United Kingdom.

**Fig. 1:**
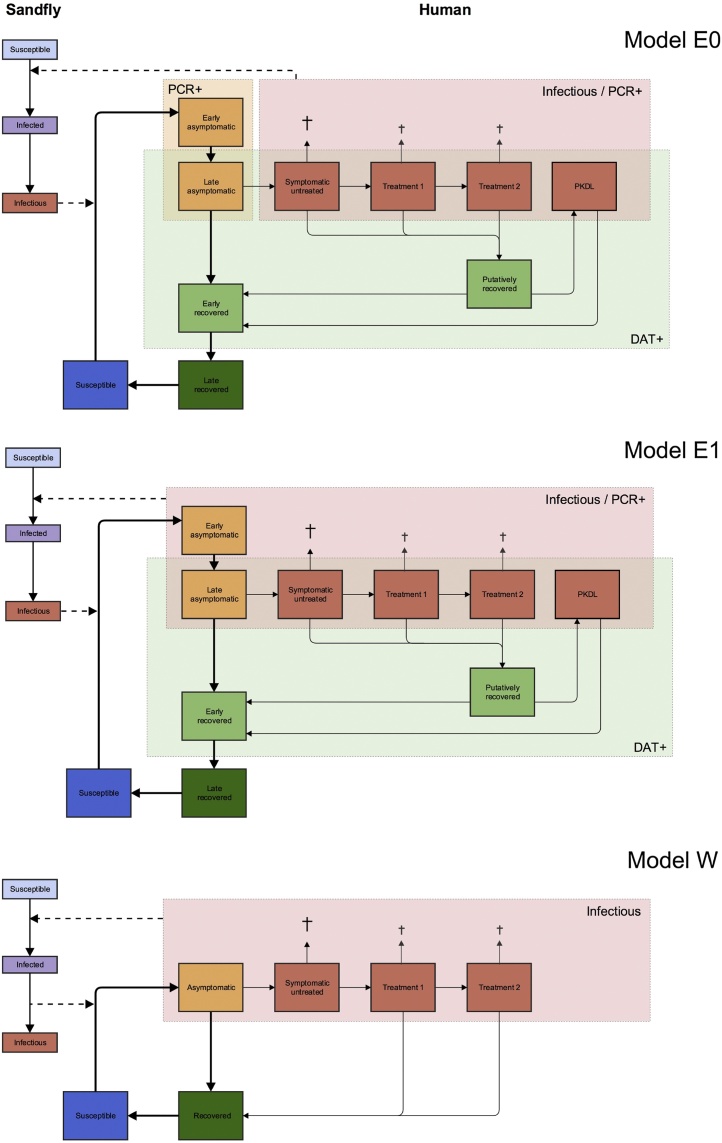
Schematic representation of model structures. Model E0 (Erasmus MC) assumes only symptomatic individuals (red boxes) are infectious towards the sandfly. In model E1 (Erasmus MC) asymptomatic individuals (yellow boxes) are the main contributors to transmission. The red shaded frame includes individuals that tested positive for parasite DNA on a polymerase chain reaction test (PCR+) and the green shaded frame includes individuals that tested positive for anti-leishmanial antibodies on the direct agglutination test (DAT+), obtained from the KalaNet study. In model W (Warwick University), asymptomatic individuals are the main contributors to transmission.

Erasmus MC developed two VL models: model E0, in which symptomatic individuals are the sole contributors to transmission, and model E1 (the best-performing model from their recent study (Le Rutte et al., 2016)) in which the main contributors to transmission are asymptomatic individuals. Supplementary File 4 contains guidelines to run the R-package ‘VLode’ of their age-structured system of ordinary differential equations for visceral leishmaniasis transmission, which is provided in Supplementary File 5. Warwick developed model W, which converts their recent Markov model of the natural history of VL (Chapman et al., 2015) into a transmission model with vector population dynamics, in which asymptomatic individuals are the main contributors to transmission. The MATLAB code for Warwick model fitting and predictions is provided in Supplementary File 6. A schematic presentation of the three models (E0, E1 and W) is given in Fig. 1 and the main model characteristics are listed in Table 1. The models are all deterministic compartmental transmission models inspired by an earlier VL transmission model developed by Stauch et al. (Stauch et al., 2011, Stauch et al., 2014). In the models, susceptible humans can become asymptomatically infected when bitten by an infectious sandfly. The majority of infected humans recover without developing clinical symptoms and only a small proportion develop clinical VL. Symptomatic cases can receive one or two VL treatments, and in models E0 and E1 treated individuals can develop PKDL after some period of apparent, but not absolute recovery (this putatively recovered stage is included because some VL cases seem to harbor dormant infection after treatment which leads to this late post-treatment dermatological complication of VL (Ramesh et al., 2015)). Susceptible sandflies become infected when they bite infectious humans (who may include asymptomatically infected individuals, symptomatic cases, treated individuals and PKDL cases), and remain latently infected for some period before they become infectious to humans. As an important new aspect, relative to previous VL transmission models, all models presented here include seasonality in the sandfly density, an important feature in VL transmission dynamics on the ISC (Singh and Singh, 2009). The differences between the models are described in detail below.

#### 2.1.1 Erasmus MC models (E0 and E1)

The set of Erasmus MC VL transmission models is defined in terms of a system of ordinary differential equations (ODE) and has been previously described and fitted to data from India and Nepal (Le Rutte et al., 2016), collected through the KalaNet study (Picado et al., 2010a). The models include population growth of both humans and sandflies (the populations are assumed to grow at the same rate in the absence of seasonality and IRS) and age-structure in human mortality and exposure to sandflies. In contrast to the Warwick model, there are compartments for early and late asymptomatic infection, and early and late recovered stage, to allow the fitting of these models to prevalence of positivity on the direct agglutination test (DAT) and/or PCR from the KalaNet study.

In this study, models E0 and E1 have been extended with a yearly seasonal pattern in sandfly density based on seasonal patterns observed in sandfly distribution studies in Bihar (Tiwary et al., 2013, Poché et al., 2011, Malaviya et al., 2014a, Picado et al., 2010b). Seasonality is implemented via a stepwise function in the sandfly birth rate, which is assumed to peak during 3 months of the year (July–September) (Poché et al., 2011). Full details of the model, including the description, characterization and calculations of equilibria of the system of ordinary differential equations along with data are provided in Additional File 1 of Le Rutte et al. (2016).

#### 2.1.2 Warwick model (W)

The Warwick VL model (Fig. 1) is very similar in structure to the models of Stauch et al., 2011, Stauch et al., 2014 and Le Rutte et al. (2016), but with some key simplifications which have been made for parsimony, given the available data, and to provide a contrast to the Erasmus MC models. The main difference is that it does not include PKDL and dormant infection (in which the individual still harbors a small number of parasites but is no longer infectious) following asymptomatic infection or VL treatment. Pathways for disease progression in the models are essentially the same, but there is only one compartment for asymptomatically infected individuals instead of two (for early and late stages), and recovered individuals are not differentiated by whether they have recovered from asymptomatic or symptomatic infection, or whether they are still seropositive or not (they are all combined into one recovered class). The latter simplification is made as the model is only fitted to the CARE data, which contains no information on individuals’ serological status, and because VL patients’ parasite loads decrease rapidly following successful treatment (Verma et al., 2010, Sudarshan et al., 2011), supporting the view that sandfly infection rates from treated patients and individuals who have recovered from asymptomatic infection are both negligible, as assumed in the Stauch and Erasmus MC models. The compartmental structure of the human part of the model is inspired by that of the Markov model developed by Chapman et al. (Chapman et al., 2015). As individuals’ onset-to-treatment (OT) times are included in the data, Erlang distributions (Gamma distributions with integer-valued shape parameters) are fitted to the OT time distributions for each district, and different numbers of compartments for clinical VL used for each district according to the shape parameter of the Erlang distribution (Mubayi et al., 2010). The model also includes seasonality in the sandfly population via sinusoidal forcing of the sandfly birth rate, such that the sandfly population varies seasonally about a constant mean. A constant human population is assumed (so the mean sandfly-to-human ratio (SHR) remains constant), and the model does not include human age-structure. Full details of the model, including the differential equations used to describe the transmission cycle and the seasonality function, are given in Supplementary File 1 (SF1).

### 2.2 Data

**Fig. 2:**
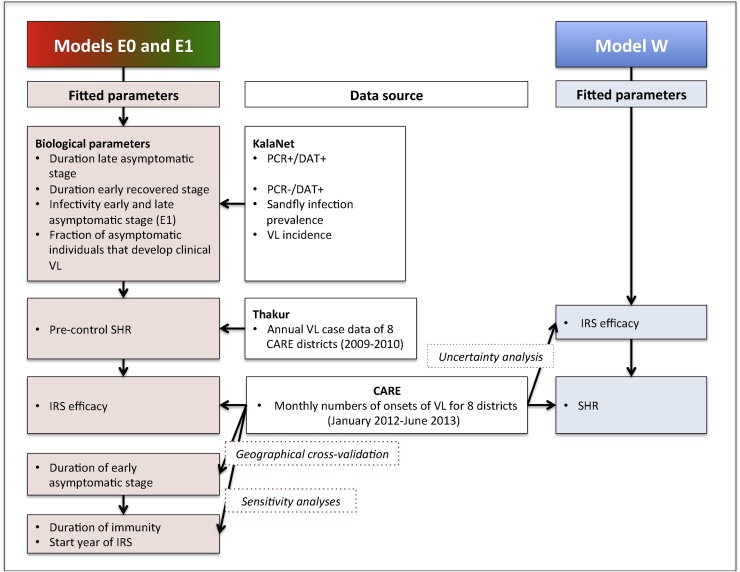
Schematic representation of the fitting procedure used by the two groups, representing the data in the white boxes and fitted parameters per model. SHR = sandfly-to-human ratio, IRS = indoor residual spray.

Both teams used longitudinal data on numbers of identified cases from the CARE study: Erasmus MC to fit the IRS efficacy (the factor that together with the IRS coverage determines the impact of IRS on the SHR) (E0 and E1) and Warwick to fit the SHR (W). Models E0 and E1 were also fitted to serological population data from the KalaNet study (Picado et al., 2010a) to estimate biological parameters, and to additional epidemiological data (Thakur et al., 2013) to estimate the pre-control SHR. An overview of the CARE, KalaNet and additional epidemiological data and the fitting process for each model is provided in Fig. 2.

#### 2.2.1 Identified cases

In 2013, CARE India collected data on 6081 VL cases in 8 districts in Bihar – Saharsa, East Champaran, Samastipur, Gopalganj, Begusarai, Khagaria, Patna and West Champaran – for an 18-month reference period for their diagnoses from January 2012 to June 2013. The cases were mainly identified from the medical records of public health facilities (primary healthcare centres, and sub-district and district hospitals), though roughly 15% were referred by other patients, relatives, accredited social health activists and private labs and doctors/hospitals. The house of each case was visited and the VL patient or a relative was interviewed twice to obtain information about the case. Individual-level data collected included the following, all of which are used in this study: age, district, sub-district, date of onset of symptoms, date of diagnosis, times from onset to first and (if applicable) second VL treatment, type and duration of treatment, occurrence of a relapse, PKDL, and details of IRS spraying in the patient’s house and neighborhood. All data were anonymized after collection. Also, data about various socio-economic factors, such as caste, house type and construction, and cattle ownership were collected. These data are not used for our study but are described in more detail by Jervis et al. (Jervis et al., 2017). Table S1 in Supplementary File 2 (SF2) shows the burden of identified VL cases for each district, and Fig. S1 in SF2 the monthly numbers of onsets of VL symptoms in each district from January 2012 to June 2013. The latter is the data from the CARE study to which the three models are fitted. A seasonal pattern in onset of symptomatic VL cases can be identified, comparable to what has been found in other VL datasets (Malaviya et al., 2011). Further description of the CARE data and how it is used to parameterize the models is provided in SF2.

#### 2.2.2 Additional epidemiological data

The KalaNet dataset includes age-structured data on DAT and PCR prevalence and conversion (from negative to positive and vice-versa) as measured in repeated surveys as well as VL incidence data monitored continuously amongst 21,267 individuals in Bihar, India, and Nepal from 2006 to 2009, and infection prevalence in the sandfly population in Nepal (Picado et al., 2010a). PCR data were collected in a sub-population of individuals aged 15 years and older.

Longitudinal epidemiological data presented by Thakur et al. (Thakur et al., 2013) on the VL case burden in the 8 CARE districts show an equilibrium situation of approximately 3 years from 2009 to 2011, before a decline in the number of cases in 2012 towards those in the CARE data (see Fig. S2 in SF2). These data were therefore taken as pre-control data on the VL case burden (i.e. data from before good quality IRS implementation and reductions in delays to treatment). The average of the annual number of cases per district in 2009 and 2010 was used to estimate the pre-control sandfly-to-human ratios for all 8 districts for models E0 and E1. Here, the model predictions for the incidence of ‘symptomatic treatment 1′ stage were fitted to the data (since the data consisted of numbers of treated VL cases). This was different for the CARE dataset, for which the model predicted incidence of ‘symptomatic untreated’ cases was fitted (as the data comprised numbers of onsets of VL symptoms). For further details see Supplementary File 2.

Parameter values that could not be taken from the CARE data, KalaNet data, Thakur data or the 2011 Indian Census were obtained from the available relevant literature.

### 2.3 Model fitting and comparison

A geographical cross-validation approach was used to test the models’ abilities to predict the monthly number of cases in each district in the CARE data over the 18-month reference period (January 2012–June 2013). This consisted of censoring the data for one district, fitting the model to the monthly numbers of VL onsets in the other 7 districts, and predicting the trend in the monthly numbers of onsets in the censored district, and then repeating the process with a different district censored. Different approaches were used by Erasmus MC and Warwick to estimate the case numbers in the censored district. Erasmus MC (models E0 and E1) fitted the IRS efficacy jointly across the 7 uncensored districts and used this as the estimate of the IRS efficacy in the censored district together with the pre-control district sandfly-to-human ratio (SHR) estimated from the Thakur data. Warwick (model W) first estimated the IRS efficacy for all 8 districts via a parameter uncertainty analysis (see SF1), then fitted the SHRs for the 7 uncensored districts and estimated the censored district SHR by linear regression of the 2012 average district identified case burdens (Table S1 of SF2) on the fitted SHRs (see Supplementary File 3 (SF3)). Other data from the censored district that were used to predict the monthly number of onsets included the mean OT time for 2012 and 2013, and the 2012 IRS coverage level. The OT time prior to 2012 was assumed to be the same as in 2012. In all models, IRS was assumed to have started in January 2011 across all districts based on there being significant increases in the reported coverage of IRS with DDT (dichlorodiphenyltrichloroethane) between 2008/2009 and 2011/2012 (from as low as 12–17% in 2008/2009 to as high as 80–91% in 2011/2012 (Malaviya et al., 2014b, National Vector Borne Disease Control Programme, 2012, Hasker et al., 2012)). All district specific information is provided in Table S1 of SF2. The three models are compared on their ability to fit the VL case data for the 8 (censored) districts of the CARE dataset with the geographical cross-validation approach, using the deviance (twice the difference in the negative log-likelihoods of the fitted model and a saturated model that fits the data exactly) between the 18 data points per district and the model output.

One of the purposes of this study was to evaluate the predictive power of the models given surveillance data, if these were available in real time, and, vice versa, to evaluate whether routine surveillance data, together with models fitted to historic data, can provide enough information from which to infer key model parameters. Therefore two approaches were taken. Warwick only used the case data to fit their model, whereas Erasmus MC included additional data to provide more information to fine-tune key epidemiological parameters. Below we describe the specific fitting approaches of both groups, a flow diagram of which is presented in Fig. 2.

#### 2.3.1 Erasmus MC VL-models

##### 2.3.1.1 Estimation of biological parameters

Model E1 has previously been fitted to the KalaNet dataset. This resulted in a relatively long estimated duration of the early asymptomatic stage of 382 days. This long duration dampens the effect of seasonal sandfly abundance on seasonality in VL incidence and therefore seems less plausible than previously thought, given the strong seasonal pattern in the newly available CARE dataset (seasonal patterns in equilibrium for different durations of early asymptomatic stage are illustrated in Fig. S3 of SF3, years 2010–2011). Because the long duration of the asymptomatic stage was primarily driven by the use of both PCR prevalence and incidence data in our previous fitting exercise, here we no longer used the PCR incidence data and assumed a series of 10 pre-set values for the duration of the early asymptomatic stage (PCR+/DAT-, of between 112 and 382 days), of which the longest value is the estimated duration in the previous study. The 20 sub-models (Models E0 and E1 each with 10 durations of the early asymptomatic stage) were refitted to the KalaNet dataset to re-estimate the following biological parameters: the duration of the late asymptomatic stage (PCR+/DAT+), the duration of the early recovered stage (PCR-/DAT+), the infectivity of the early and late asymptomatic stage (model E1 only), and the fraction of asymptomatic individuals that develop clinical VL (VL incidence). The age-dependent exposure to sandflies and the duration of the late recovered stage of two years (immunity) were chosen based on the conclusions from the previous paper. A sensitivity analysis was performed in this study to also explore the impact of assuming durations of immunity of one and five years. All literature based parameter values can be found in Table 2, Table 3, and all fitted parameters in Table 4 of the results section.

##### 2.3.1.2 Sandfly-to-human ratio (SHR)

The 20 sub-models were then fitted to the average VL incidence of 2009 and 2010 from Thakur et al (Thakur et al., 2013), which we interpreted as an equilibrium due to the stability of incidence over the given years in the data, to estimate the district-specific SHR in the absence of IRS.

##### 2.3.1.3 IRS efficacy

The IRS efficacy was estimated by fitting each model to the CARE data on the trend in the numbers of cases over the 18-month period. Within the model, the IRS impact on the sandfly birth rate was defined as the IRS efficacy multiplied by the district-specific IRS coverage rate reported in the CARE data (i.e. the sandfly birth rate was multiplied by 1 minus the aforementioned product). A sensitivity analysis for the start year of IRS was performed by fitting the model with IRS starting in 2010 and in 2012.

##### 2.3.1.4 Parameter estimation

All parameters were estimated by maximum likelihood estimation using the BFGS algorithm from the optim package in R (version 3.3.0). The data-generating process for prevalent cases (DAT and PCR positivity) was assumed to be a binomial distribution, and that for incident VL cases (VL cases) a Poisson distribution. We calculated confidence intervals for biological parameter estimates based on the inverse of the Hessian at the global optimum (i.e. assuming a multivariate normal distribution). We further calculated the deviance between each sub-model and a hypothetical, saturated model exactly predicting the data. Based on the deviance, we selected the sub-models with the duration of early asymptomatic stage that was closest to the saturated model (i.e. lowest deviance) for both model E0 and E1. The sub-models best fitting the data were then used to generate further predictions. More details of the model fitting procedure are presented in Supplementary File 3.

#### 2.3.2 Warwick VL-model

##### 2.3.2.1 Biological parameters

Values of biological parameters such as the average duration of asymptomatic infection and duration of immunity were based on Chapman et al’s (2015) modelling of the natural history of infection. Other parameter values (for disease progression and sandfly bionomics) were based on estimates from previous field and modelling studies (see Table 2, Table 3). A parameter uncertainty analysis was performed to estimate the uncertainty in key transmission parameters (see below).

##### 2.3.2.2 Sandfly-to-human ratio (SHR)

Model W was fitted to the CARE data by estimating the average SHR for each district by maximum likelihood estimation. The number of new cases in each month was assumed to be Poisson distributed, and the full likelihood for each district taken as the product of the individual probabilities of the monthly numbers of cases predicted by the model.

##### 2.3.2.3 IRS efficacy

IRS was assumed to increase the sandfly death rate by a percentage equal to the product of the IRS efficacy factor and the district IRS coverage rate from the CARE study. The model was fitted both with a fixed IRS efficacy factor (chosen based on the parameter uncertainty analysis) and assuming no impact of IRS on the sandfly density, and the goodness of fit compared using the Akaike Information Criterion (AIC = 2k − 2log(L), where k = 8 is the number of estimated parameters and L is the total likelihood for the 8 districts) to assess whether IRS may have had a significant impact on VL transmission.

##### 2.3.2.4 Parameter uncertainty analysis

Given the high degree of uncertainty in the values of several of the model parameters, a parameter uncertainty analysis was carried out to determine 95% confidence intervals for 9 parameters: the SHR, the amplitude and phase shift of the seasonal variation in the sandfly birth rate, the proportion of asymptomatic individuals who develop clinical VL, the average duration of asymptomatic infection, the relative infectivities of asymptomatic individuals and clinical VL cases, the duration of immunity and the IRS efficacy factor (see SF1). Confidence intervals were determined by simulating the model with 300,000 parameter sets sampled from random uniform distributions for the parameters and accepting those for which the likelihood was not significantly different from the maximum likelihood according to the likelihood ratio test. Further details of the model fitting method and parameter uncertainty analysis are provided in SF1.

### 2.4 Predictions of future VL trends

We first predict the trends in VL incidence in the 8 CARE districts up to 2020 assuming the continuation of the district-specific 2012 intervention levels, i.e. the same IRS coverage and OT time. Then we explore hypothetical scenarios with incidence rates of 10, 5 and 2 VL cases per 10,000 capita per year, since the estimated VL endemicities at sub-district level from the CARE data in Bihar in 2012 ranged between 0 and 9.1 VL cases per 10,000 capita per year. The distribution of sub-district incidences can be found in Fig. S4 of Supplementary File 2. For these hypothetical scenarios, VL incidence is predicted for the default scenario of 60% IRS coverage and 40-day average OT time, and scenarios with an increased IRS coverage of 80% and shorter average OT time of 20 days to reflect a further improvement of the control program.

## 3 Results

### 3.1 Parameter estimation

Table 4 presents the values of all fitted parameters of models E0, E1 and W. With a duration of the early asymptomatic stage of 112 days and shorter the ability of the models E0 and E1 to reproduce the KalaNet data rapidly declined. The best fitting sub-models had a duration of early asymptomatic stage of 202 days (deviances of all 18 stable sub-models are presented in Table S3 of SF3). With model E0, the pre-control SHR ranged from 1.84 in West Champaran to 3.92 in Saharsa and with model E1 from 0.35 in West Champaran to 0.60 in Saharsa. The IRS efficacy is estimated by fitting the two models to all available CARE data points from the 8 districts simultaneously and resulted in a value of 99.9% (Model E0) and 82.9% (Model E1), which corresponds to a sandfly birth rate reduction of between 49.7% and 59.9% when multiplied by the average district IRS coverage rate of 60%. The results and interpretation of the sensitivity analyses for the duration of immunity (1 and 5 years) and the start year of IRS (2010 and 2012) are presented in Tables S4–7 of SF3. These results suggest that the duration of immunity is probably close to 2 years, as was assumed in the previous study (Le Rutte et al., 2016). Further, the selected start year of IRS in 2011 fitted the data best.

For model W, an average IRS efficacy value of 0.006, equivalent to an annual reduction of 9% in the sandfly density with an IRS coverage of 60%, was found to give the best fit to the data from a range of values tested in the parameter uncertainty analysis. The large discrepancy between the fitted IRS impact of models E0 and E1 (50–60%) and model W (9%) is largely due to the Erasmus MC models also including the drop from the Thakur data to the CARE data in their fitting procedure. Besides this, a greater IRS impact is also required for models E0 and E1 to reach the short term drop in cases, due to the longer duration of the asymptomatic stages (270 days in models E0 and E1 versus 150 days in model W). The district average SHRs that were estimated from fitting model W to the CARE data for each of the 8 districts individually (without censoring) are also presented in Table 4. The SHRs are positively correlated with the district average identified case burdens (compare Table 4 with Table S1 in SF2 and see Fig. S1 in SF3), ranging from 0.36 in West Champaran to 0.45 in Saharsa. When model W was fitted under the assumption that IRS had no impact on incidence, the model was unable to reproduce the drop (in all districts but West Champaran) between the first peak in the number of cases in 2012 and the second peak in 2013 (results not shown). The model only predicted a very slight drop in the number of cases in each district due to the decrease in the OT times from 2012 to 2013. Varying the change in the mean OT time for each district showed that an approximately 10-fold decrease in the time to treatment would be required to produce the drop in the numbers of cases from 2012 to 2013, a far greater decrease than observed in the actual OT times for any of the districts. The AIC value for the model with the IRS efficacy factor of 0.006 taken from the parameter uncertainty analysis was significantly lower than for the model with no effect of IRS (AIC = 1082.2 compared to AIC = 1400.8). This suggests that the drop in the numbers of cases across the districts was more likely due to decreases in the sandfly populations, which may have occurred as a result of IRS or other extraneous factors, than decreases in the OT times. However, we note that these results are dependent on the assumed infectivity of asymptomatic individuals (such that they are the main contributors to transmission) and the estimated efficacy of IRS.

### 3.2 Geographical cross-validation

**Fig. 3:**
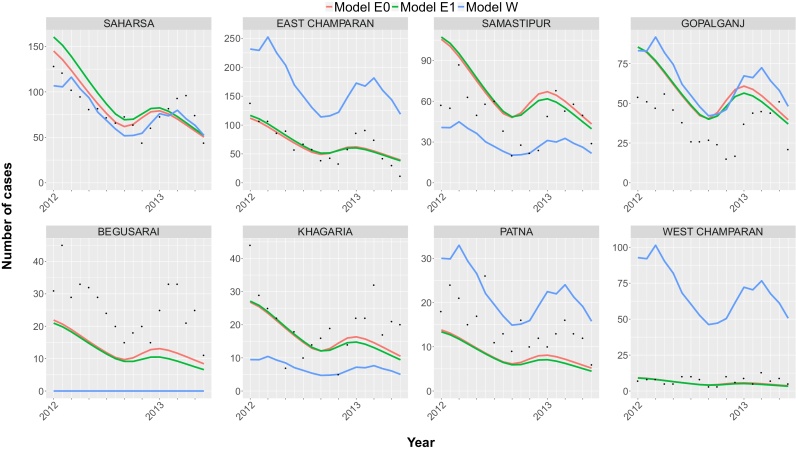
Geographical cross-validation of the models: predictions for the monthly number of VL cases in each district from fitting the models to the other 7 districts and using the fitted models to predict the cases in the censored district. The CARE data are presented with black dots and the lines present the predictions of models E0 (red), E1 (green) and W (blue) between January 2012 and June 2013. The prediction of 0 cases for Begusarai for model W is due to the sandfly-to-human ratio estimated from fitting to the other 7 districts giving a basic reproduction number below 1 (so that there is no stable endemic equilibrium).

The monthly VL case numbers for each censored district predicted by the three models in the geographical cross-validation, together with the observed case numbers from the CARE dataset are presented in Fig. 3. Models E0 and E1 predict the data of censored districts Saharsa, East Champaran, Khagaria and West Champaran relatively accurately. However, in Begusarai and Patna the models both predict slightly lower numbers of identified cases compared to the data and slightly higher numbers in Samastipur and Gopalganj. For model W, the SHR for the censored district was informed solely by the average case burden in 2012 in the censored district, and therefore the predictions for the censored districts are less accurate than for models E0 and E1, which are informed by multiple data points from historical data. As expected given the simplicity of the estimation method, the fits are better when all the districts are uncensored (compare Fig. 3 with Fig. S2 in SF3) and the method suffers from considerable inaccuracy for the lowest burden district (West Champaran). For Begusarai, the SHR estimated from linear regression of the 2012 average district case burdens on the fitted SHRs for the other 7 districts is insufficient to give a stable endemic equilibrium (i.e. the method predicts zero cases).

### 3.3 Prediction of elimination

**Fig. 4:**
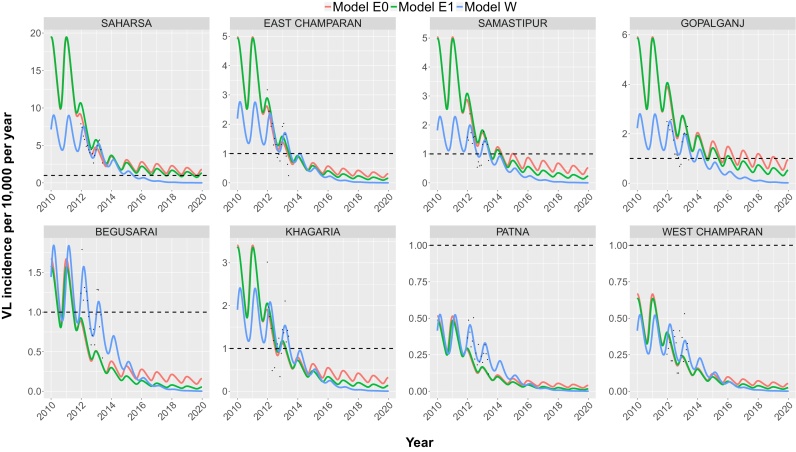
VL incidence predictions for each district employing all of the CARE data. The CARE data are presented with black dots and the lines present the predictions between 2010 and 2020 for each model. IRS starts in January 2011, after which IRS coverage remains constant. The difference in the pre-control endemic equilibrium between the Erasmus MC and Warwick models is due to Erasmus MC fitting to historical case data from (Thakur et al., 2013) before fitting to the CARE data and Warwick fitting only to the CARE data. The black dashed line represents the WHO elimination target of <1 VL case per 10,000 population per year. The monthly predictions between January 2012 and June 2013 are also presented in SF3, Fig. S2.

Fig. 4 presents forward predictions of VL incidence up to 2020 for all 8 districts with their district-specific IRS coverage (2012) and OT time (2013). Here, the three models were fitted to all of the available CARE data. Models E0, E1 and W all show that the target incidence of <1 VL case per 10,000 capita per year will be, or has already been, reached at district-level in all districts before 2020, apart from Saharsa as predicted by models E0 and E1. In fact, for Patna and West Champaran the data already indicate an identified VL case burden in 2012 below the target of <1 new VL case per 10,000 population per year. Due to the seasonal variation in incidence, the predicted incidence for the other districts oscillates above and below 1 VL case per 10,000 capita per year before decreasing and remaining below the target. We interpreted the final point at which the model predictions pass through the target incidence line as the district-level elimination time. Models E0 and E1 predict that Begusarai, Khagaria, East Champaran, Samastipur and Gopalganj will reach the elimination target between mid-2011 (E1) and 2019 (E0). Model E0 predicts a slower trend towards elimination compared to model E1, with elimination occurring a maximum of 3 years later (Gopalganj), due to the slower impact of interventions caused by symptomatic cases (VL and PKDL) being the main contributors to transmission as opposed to asymptomatic individuals. The difference in predictions of reaching the elimination target between the shortest (142 days) and longest (382 days) duration of the early asymptomatic stage, ranges between 1 and 3 years, depending on the district, with the longest duration until elimination predicted by the model with the longest duration of the early asymptomatic stage (Fig. S3 in SF3). Model W predicts that Begusarai, Samastipur, Khagaria, East Champaran and Gopalganj reached the target between April 2013 and May 2014 (in that order), and Saharsa in June 2015. The systematic difference in the pre-control endemic equilibrium between models E0 and E1 and model W is due to Erasmus MC fitting to historical annual case data from Thakur et al. (2013) before fitting to the CARE data and Warwick only fitting to the CARE data.

**Fig. 5:**
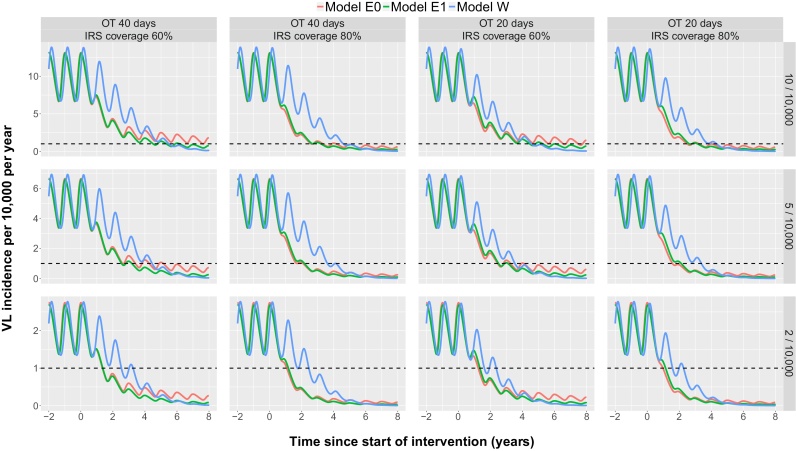
VL incidence predictions at sub-district level for different endemic scenarios under alternative intervention strategies. The lines present the VL incidence predictions for each model. The black dashed line represents the WHO elimination target of <1 VL case/10,000 population/year at sub-district level. Interventions start in year 0 after which they are continued at the same level. OT denotes the time between the onset of symptoms and the start of treatment, IRS coverage represents the percentage of houses sprayed, which is multiplied by a (constant) IRS efficacy of 0.999 and 0.829 in models E0 and E1 and 0.006 in model W. The 12 predictions presented here include 3 different endemic scenarios of 10, 5 and 2 cases per 10,000 population each with 4 different combinations of interventions.

As described above, the WHO elimination as a public health problem goal is defined as reaching the target at sub-district level, not at district level. The predicted incidences for three typical sub-districts with current and alternative intervention strategies are presented in Fig. 5. In sub-districts with a pre-control endemicity level of 2/10,000, models E0 and E1 predict that elimination will be reached under the current interventions (an average OT time of 40 days and 60% IRS coverage) ∼1.5 years after starting IRS, whereas under the same conditions model W predicts that elimination will be reached after 3.5 years. In sub-districts with a pre-control endemicity of 10/10,000, model W predicts reaching the target incidence after 5.5 years, model E1 after 6 years and model E0 after more than 8 years from the start of IRS. Increasing the IRS coverage from 60% to 80% or halving the OT time from 40 days to 20 days brings forward elimination by 6 months to 3 years at all endemicity levels for all models (the reductions in the time to elimination being greater for the sub-districts with higher pre-control endemicities). Models E0 and E1 predict that increasing the IRS coverage to 80% will have a greater effect than halving the OT time, whereas model W predicts that halving the OT time will have a slightly greater effect. However, combining an increase in IRS coverage to 80% with a reduction in OT time to 20 days is predicted to reduce the time to elimination at all endemicity levels for all models.

## 4 Discussion

The Erasmus MC and Warwick models are quite different in their model structure, fitting methodologies and use of data, yet their long-term predictions are comparable and suggest that elimination can be reached by or shortly after 2020 even in highly endemic sub-districts (up to 10 VL cases per 10,000 capita per year pre-IRS), provided IRS started before or in 2011 with a minimum coverage level of 60% and is maintained at this level, and provided that the average onset-to-treatment (OT) time does not exceed 40 days. In settings with a pre-control endemicity level of 5/10,000, increasing the effective IRS coverage from 60 to 80% is predicted to lead to elimination of VL 1–3 years earlier (depending on the type of model), and decreasing OT from 40 to 20 days to bring elimination forward by approximately 1 year.

All VL transmission models face the challenge of describing transmission of a disease with many key factors, such as the role of immunity and the contributions of individuals in different disease stages to transmission, basically remaining unknown (Rock et al., 2015, Rock et al., 2016, Le Rutte et al., 2016). Also the impact of IRS, the main intervention strategy, on sandfly densities remains debated. Together, the KalaNet and CARE studies provide information about the prevalence of leishmania DNA and anti-leishmanial antibodies in the population, as well as detailed data on start of symptoms, detection and treatment dates of symptomatic individuals. Since both studies were conducted in the same region, biological parameters estimated from the KalaNet data were considered to be similar in the CARE study sites, but this is not necessarily the case. Also, although longitudinal, the CARE dataset only covered a time period of 18 months, which is short relative to the long duration between time of infection and cure of the disease, the time until development of PKDL and the potentially long duration of immunity. The sensitivity of the sub-district-level model predictions to the estimated reduction in the sandfly-to-human ratio due to IRS (9% for model W, 50% for model E0 and 60% for model E1) demonstrates the importance of collecting sandfly data alongside human epidemiological data to properly quantify the impact of IRS. Furthermore, the CARE dataset did not provide information about the start years of IRS per district, sandfly bionomics data from the same location and time period as the VL case and IRS coverage data, or sandfly DDT-resistance data, all important factors to estimate the impact of IRS strategies on VL incidence. Given the relatively long duration of the asymptomatic infection stage, the duration of immunity and the role of PKDL (which could slow down the initial decline seen in the first two years of control (Le Rutte et al., 2016)), the decline in VL incidence due to IRS is expected to be seen only months to years after the start of IRS; hence data spanning a longer time frame would be valuable for future studies.

Both model E0, in which asymptomatic individuals are not infectious, and model E1, where the main contributors to transmission are asymptomatic individuals, provided an estimate of about 200 days as the duration of the early asymptomatic stage best fitting the CARE data (i.e. 9 months when including the late asymptomatic stage). For model W, the average duration of asymptomatic infection was based on (Chapman et al., 2015), where it was estimated as approximately 5 months (95% CI 4–5.5 months). This estimate was used in this paper as it is supported by the presence of a seasonal pattern in the monthly numbers of VL cases in the CARE districts and the timing of this pattern relative to observed seasonal variation in sandfly abundance in Bihar (Poché et al., 2011, Malaviya et al., 2014a, Picado et al., 2010b, Kesari et al., 2014). It also agrees reasonably well with the results of the parameter uncertainty analysis for model W (the maximum likelihood estimates for the asymptomatic infection duration for the 8 districts ranged from 98 to 163 days, see SF1). Even though the main contributors to transmission remain unknown, the predictions of models E0 and E1, which both fit the data nearly equally well, provided new insights in reaching the elimination targets in both (extreme) scenarios. Model W is comparable to model E1 in terms of asymptomatics being the main contributors to transmission, but because of differences between the models in the durations of disease stages and fitting the models to different data, the fitted IRS impact for model W is much lower, leading to a slower reduction in incidence with current and improved interventions. When more information becomes available regarding the main contributors to transmission, more weight can be put on the predictions of model E0 or model E1 (or model W), which differ most in terms of long-term trends.

Another unknown aspect of the VL dynamics is the role and duration of immunity. We therefore repeated our analyses of models E0 and E1 for two alternative durations of 1 and 5 years. With a 5-year average immunity it was not possible to fit the sub-models to the data with an early asymptomatic stage duration shorter than 322 days (which was one of the 10 chosen durations). However, in our study, we interpreted the KalaNet and Thakur data as endemic equilibriums, which is not always the case in the field when looking at longitudinal data, especially at local level. We might have arrived at a different duration of immunity had we been able to fit our sub-models to data including fluctuations in incidence over multiple years at a local level (Bora, 1999, Muniaraj, 2014, Thakur, 2007). We also performed a sensitivity analysis of models E0 and E1 for the start year of IRS, for which no conclusive data were available. We interpreted the start year of IRS in 2011 to be the most likely scenario when compared to starting IRS in 2010 or 2012, because of the decrease in VL incidence in multiple datasets after 2011 (Thakur et al., 2013, State Surveillance Unit, 2012; Independent Commission on Development & Health in India, 2014), and since starting IRS in 2012 fitted the data poorly (see SF3).

The fitted IRS efficacy for models E0 and E1 led to a sandfly birth rate reduction between 37% in Gopalganj and 72% in Saharsa, which aligns with reported reduction in sandfly density of 72% after IRS with DDT (Joshi et al., 2009). For model W, the IRS efficacy corresponds to an annual reduction in sandfly density of 6.7% in Gopalganj and 10.7% in Saharsa. This is apparently at odds with estimates for reductions in sandfly densities from field studies (Joshi et al., 2009), but could reflect poor implementation of IRS and increasing sandfly resistance to DDT (Coleman et al., 2015). However, given the correlation of the IRS efficacy factor with other parameters (e.g. the average duration of asymptomatic infection), it is also possible that the IRS efficacy factor has been underestimated due to inaccuracies in the estimates for correlated parameters. The issue of parameter identifiability is not restricted to the IRS efficacy, as the parameter uncertainty analysis for model W in SF1 shows. The average sandfly-to-human ratio and the infectivity of asymptomatic individuals are strongly negatively correlated, and the average duration of asymptomatic infection and amplitude of the seasonal forcing of the sandfly birth rate are positively correlated for most districts, which means that these parameters cannot be uniquely identified from the CARE data, which may account for some of the differences between the models’ predictions. Hence, data from high quality epidemiological and entomological studies are needed alongside the CARE data to tease apart the correlation between these parameters. Using more detailed data on disease progression (e.g. longitudinal serological data for humans and infection prevalence data for sandflies) would then also improve the accuracy of model predictions (as demonstrated by models E0 and E1).

In model W it was assumed that 3% of asymptomatic individuals develop clinical symptoms even though the estimated proportion for the dataset analyzed in (Chapman et al., 2015) was 14.7% (95% CI 12.6–20.0%). This is because the maximum proportion that the CARE data can support is 7.3%, assuming an asymptomatic prevalence of approximately 1% at district level and a long asymptomatic duration of 530 days (Le Rutte et al., 2016) (see SF1), and because more recent studies from Bihar suggest values in the region of 3–4% (Hasker et al., 2014). This difference in the estimated proportion of asymptomatics progressing to VL may reflect the different locations and time periods in which the studies were conducted – the former in Bangladesh during an epidemic, the latter in Bihar, India, 4–5 years after an epidemic (Hirve et al., 2016). However, due to the focal nature of VL it is also likely that we have significantly underestimated local VL incidence from the CARE dataset that all three models were fitted to, because we calculated the identified case burden at district-level, such that a higher proportion of asymptomatic individuals progressing to VL is likely.

Another potential source of inaccuracy in model W is the omission of PKDL. Including PKDL in the model results in longer times to elimination, as there is a reservoir of infectious individuals with potentially long durations of infectivity. However, PKDL was excluded due to uncertainty regarding the disease history of PKDL cases and the proportion of individuals with PKDL that were actually diagnosed.

All models currently assume that there is homogeneous risk of transmission, but VL occurrence is highly spatially heterogeneous and at a scale much smaller than that of a district (Bern et al., 2010, Bhunia et al., 2013). Consequently, we are averaging the incident cases over the total population of all affected sub-districts regardless of how many of those people were at risk of infection, thus underestimating the incidence in those actually at risk. Models with finer geographic stratification (e.g. at the level of villages or even sets of households) and migration would present a more realistic framework and potentially provide an important tool for modelling elimination of infection. An individual-based model would be able to include advanced aspects such as these. Less than half of the 8885 villages and town wards in the 8 districts had a VL case during the study period. However, there may be significant transmission in villages without cases during the timeframe of the data if the asymptomatic to symptomatic ratio is as high as estimated, but this cannot be discerned from the CARE data since it contains no information on asymptomatic cases.

District- and state-level data on annual case numbers suggest that there are long-term cycles (with a period of ∼15 years) in incidence, which are not produced by our models and cannot be readily explained by them either (Bora, 1999, Muniaraj, 2014, Thakur, 2007). The models all assume that the transmission dynamics were in equilibrium prior to 2011 and we did not implement an underlying mechanism for these ∼15-year cycles in our models because there are no data explaining such a mechanism. However, it has been suggested that these cycles have been caused by intermittent control efforts (Muniaraj, 2014). The additional impact of IRS and reduced OT time over a decrease in incidence from being in a declining phase of a long-term epidemic cycle (as appears to be the current situation) is therefore unknown. The potential role that long-term immunity could play in the epidemic cycles, by causing rapid depletion of the susceptible pool in the population during an epidemic, which is then replenished by births in the inter-epidemic period, has also not been accounted for (Dye, 1992), given the assumed short durations of immunity (2–5 years). However, it remains to be shown that such a mechanism could lead to distinguishable long-term cycles at the level of districts, let alone countries and subcontinents. Longer time series, and ideally also serological and PCR data, are needed to accurately estimate duration of immunity and other key parameters.

Whilst the deterministic compartmental models presented here are suitable for understanding and predicting the longer time-scale dynamics and interactions, they are less useful for predicting the situations close to elimination or after elimination. When numbers of cases become very small stochastic effects dominate, something all three models do not account for. Given that recrudescence would have a significant public health impact if not controlled, stochastic models would be very useful. Such models could also be used to assess the effectiveness of alternative targets. For example, since VL is such a focal disease, setting targets and focusing interventions at a smaller (e.g. village) level could be a valuable next step. However, stochastic (and in particular individual-based or agent-based) models typically contain more parameters and therefore require more detailed data and/or assumptions.

Our three models differ in terms of detailed structure and parameterization. Despite these differences, the models are consistent in predicting that we are on track to reach the target elimination incidence in all 8 Indian districts – at least at district level. Several sub-districts were already below the target incidence in 2012. The 10 sub-districts with the highest endemicity were all in Saharsa and East Champaran with ≥5 VL cases per 10,000 capita per year. These sub-districts are likely to require additional efforts, such as increased IRS coverage and reduced OT time, to reach the target incidence on time.

Capturing the disease transmission dynamics of visceral leishmaniasis in a mathematical structure is a complex challenge due to the many unknown factors regarding this neglected tropical disease (Hirve et al., 2016, Cameron et al., 2016). However, combining the outcome of different mathematical modelling approaches creates a more solid foundation from which to draw conclusions about reaching the VL elimination targets for the Indian subcontinent. We caution that a model comparison such as this can provide false confidence as there are processes which are not included in the models that might be critical. In particular, all the models assume the same underlying SIRS structure (susceptible – infected – resistant – susceptible) and none of the models includes spatial heterogeneity. Nonetheless, based on the three VL transmission models, we conclude that reaching the VL elimination target of less than 1 VL case per 10,000 capita at sub-district level before or shortly after 2020 in the Indian subcontinent seems feasible under continued interventions of indoor-residual spraying and early detection and treatment of cases.

## Author contributions

Oversaw the data collection: SD, IC, SS, ECH and MCB. Conceived and designed the study: EALR, LACC, LEC, SJ, GFM, TDH and SJdV. Cleaned the data: LACC and SJ. Performed the analysis: EALR, LACC, LEC and SJ. Wrote the manuscript: EALR and LACC. Helped with interpretation of the data: MK, AD, TM, IC, GFM, SS, TDH and SJdV. Provided feedback on the modelling and reviewed the paper: LEC, GFM, TM, SS, TDH and SJdV.

## Figures and Tables

**Table 1:** Overview of the main model characteristics.

Model characteristic	Erasmus MC Rotterdam	Warwick University
Model type	Population-based (deterministic, age-structured)	Population-based (deterministic, no age structure)
Human disease states	Susceptible, early and late asymptomatically infected, symptomatically infected untreated, first-line treatment, second-line treatment, post kala-azar dermal leishmaniasis (PKDL), putatively recovered, and early and late recovered.	Susceptible, asymptomatically infected, symptomatically infected, first-line treatment, second-line treatment, recovered.
Sandfly states	Susceptible, latently infected, infectious	Susceptible, latently infected, infectious
Main contributors to transmission	E0) Symptomatic cases E1) Asymptomatic individuals	Asymptomatic individuals
Interventions considered	Vector control (IRS) and decreasing the duration of onset of symptoms to treatment (OT)	
Human demography	Per capita birth rate and age-specific mortality rates (2011)	Population based on Indian 2001/2011 census, birth and mortality rates.
Human sandfly exposure	Age-dependent, seasonal	Seasonal
Distribution of duration of states	Exponential	Exponential except for duration of symptomatic VL, for which Erlang-2 distributions were fitted to onset-to-treatment time distributions in data

**Table 2:** Parameter values and assumptions that are similar across all models.

Parameters	Value*	Source	Reported range	Source
Human parameters				
Average duration of symptomatic untreated stage (days)	District and year specific (See SF2)	CARE data	N/A	N/A
Average duration treatment 1 (days)	28	CARE data	N/A	N/A
Average duration treatment 2 (days)	28	CARE data	N/A	N/A
Average duration of putatively recovered stage (months)**	21	(Ramesh et al., 2015, Uranw et al., 2011, Rahman et al., 2010)	21–36	(Ramesh et al., 2015)
Average duration of PKDL (years)**	5	Expert opinion and (Ramesh et al., 2015)	0.5–5	(Ramesh et al., 2015, Islam et al., 2013)
Excess mortality rate among untreated symptomatic cases (per day)	1/150	Assumption		
Excess mortality rate among treated symptomatic cases (per day)	1/120	Assumption		
Fraction of failed first-line treatments	District-specific (See SF2)	CARE data	N/A	N/A
Fraction of putatively recovered cases that develop PKDL	2.5%	CARE data	2.4%–17%	(Uranw et al., 2011, Islam et al., 2013)
Infectivity of symptomatic untreated cases	1	Reference value	0.02–0.42	(Quinnell and Courtenay, 2009)
Relative infectivity of patients under treatment 1 and 2	0.5	Expert opinion	Unknown	

Sandfly parameters				
Average life expectancy of the sandfly (days)	14	(Palit et al., 2011)	10–20	
Average duration of incubation period in sandflies (days)**	5	(Sacks and Perkins, 1985)	4.7–5.1	(Hurwitz et al., 2011, Hati et al., 1984, Shortt, 1945)
Sandfly biting rate (per day)**	1/4	(Anon., 2016, Hati et al., 1984)	1/5–1/4	(Anon., 2016, Hurwitz et al., 2011, Hati et al., 1984, Palit et al., 2011)
Probability of transmission from an infected sandfly to susceptible human	1	Reference value	N/A	

* Parameter values held fixed in model fitting and predictions unless otherwise stated.

** Ranges given are for average (median) duration based on the literature. Durations treated as exponentially distributed in models.

**Table 3:** Parameter values and assumptions that differ across the models.

Parameter	Value			Source
	Erasmus MC		Warwick	
	Model E0	Model E1	Model W	
Birth rate (per 1000/yr)	Bihar specific		District-specific (see SF2)	Indian 2011 Census
Mortality rate (per 1000/yr)	Bihar specific, age-dependent		District-specific (see SF2)	Indian 2011 Census
Sandfly birth rate	Stepwise function with 3 month peak in July–Sept		Sinusoidal function with peak in Oct–Nov	(Poché et al., 2011) (E0, E1) (Picado et al., 2010b, Kesari et al., 2014, Ghosh et al., 1999) (W)
Infectivity of PKDL, relative to symptomatic untreated cases	0.5	0.5	N/A	Expert opinion
Duration of immunity (years)	2 (1 and 5 in sensitivity analysis)	2 (1 and 5 in sensitivity analysis)	5	Assumption based on (Le Rutte et al., 2016) (E0,E1) Assumption based on (Chapman et al., 2015) (W)
Average duration of PKDL (years)	5	5	N/A	Expert opinion, (Ramesh et al., 2015)
Sandfly exposure	Age-dependent	Age-dependent	N/A	(Le Rutte et al., 2016)
Duration of early asymptomatic stage (days)	202	202	Early + late = 150	(Le Rutte et al., 2016) (E0, E1) (Chapman et al., 2015) (W)
Duration of late asymptomatic stage (days)	69 days (fitted)	69 days (fitted)		
Infectivity of asymptomatic stage	0 (pre-set)	0.0114 (early) 0.0229 (late) (fitted)	0.025	Assumption based on (Stauch et al., 2011) (W)
Fraction of (late stage) asymptomatic individuals who develop VL	0.0142 (fitted)	0.0142 (fitted)	0.03	Assumption based on (Hasker et al., 2014) (W)

**Table 4:** Estimated parameter values resulting from fitting to all district data without censoring.

Parameter*		Erasmus MC		Warwick
		Model E0 (95% CI**).	Model E1 (95% CI**)	Model W
1. Fraction of late asymptomatic individuals who develop VL (%)		1.42 (1.00–1.84)	1.42 (1.00–1.84)	N/A
2. Duration late asymptomatic stage (days)		69 (49–119)	69 (49–118)	N/A
3. Duration early recovered stage (days)		237 (196–299)	236 (196–298)	N/A
4. Infectivity of early asymptomatic stage		0***	0.0114****	N/A
5. Infectivity of late asymptomatic stage		0***	0.0229 (0–0.0533)	N/A
6. District specific average sandfly-to-human ratio (SHR)	Saharsa	3.92	0.604	0.445
	East Champaran	2.20	0.392	0.381
	Samastipur	2.49	0.401	0.398
	Gopalganj	2.33	0.403	0.390
	Begusarai	2.78	0.383	0.425
	Khagaria	2.44	0.388	0.401
	Patna	2.12	0.359	0.380
	West Champaran	1.84	0.351	0.364
7. IRS efficacy***** (Employing data from all 8 districts)		0.999	0.829	0.006

NB. Values for models E0 and E1 presented here are only for the duration of early asymptomatic stage of 202 days; values for the other sub-models are listed in Table S3 of Supplementary File 3.

* Parameters 1–5 were fitted to the KalaNet data (models E0 and E1), parameter 6 was fitted to the Thakur data (models E0 and E1) and the CARE data (model W), and parameter 7 was fitted to the CARE data (models E0, E1 and W).

** Confidence interval.

*** Pre-set values, in model E0 asymptomatic individuals are considered not to be infective.

**** Not fitted, but calculated as half the infectivity of the late asymptomatic stage.

***** The IRS efficacy is multiplied by the district-specific IRS coverage rate to get the IRS impact on the SHR. Note, however, that the dependence of the SHR on the IRS efficacy is linear in models E0 and E1, but exponential in model W (see Additional File 1 of (Le Rutte et al., 2016) and Supplementary File 1).

## Appendix A Supplementary data

Supplementary File S1 Warwick model description and parameter uncertainty analysis.

Supplementary File S2 Data description.

Supplementary File S3 Fitting procedure and Erasmus MC sensitivity analyses.

Supplementary File S4 Description to Supplementary File 5.

Supplementary File S5 R-package ‘VLode’ of age-structured system of ordinary differential equations for visceral leishmaniasis transmission developed by Erasmus MC.

Supplementary File S6 MATLAB code for Warwick model fitting and predictions.
