# Supplementary material for: Elimination of visceral leishmaniasis in the Indian subcontinent: a comparison of predictions from three transmission models
Source: Epidemics. 2017 Mar;18:67–80. doi: 10.1016/j.epidem.2017.01.002 (PMC5340844; doi:10.1016/j.epidem.2017.01.002)
Supplement: Supplementary File S1 — Warwick model description and parameter uncertainty analysis. [file mmc1.pdf]

Available online at www.sciencedirect.com
ScienceDirect
Epidemics 18 (2017) 1–17
EPIDEMICS

Supplementary File 1: Warwick visceral leishmaniasis transmission model

1. Model structure

As described in the main text, the structure of the model is similar to that of Stauch *et al* [21, 22]. The key differences are that dormant infection (following asymptomatic infection or treatment for clinical VL) and PKDL are not modelled; there are fewer compartments for asymptomatically infected and recovered individuals; and symptomatic infection is split into one or more sub-compartments depending on the distribution of onset-to-treatment times in the district being modelled. We choose not to model dormant infection and PKDL due to uncertainty in the information on PKDL in the data (information on clinical VL and PKDL history was not segregated for individuals with PKDL) and over what proportion of individuals with PKDL were actually diagnosed (as some may not have sought treatment or had clinical VL prior to PKDL). According to the data only 2.5% of individuals treated for VL developed PKDL, which, if representative of the actual PKDL incidence, suggests that PKDL cases were probably not a major source of transmission during the study period. The other two differences mentioned above reflect the fact that the data does not include any information on asymptomatic infection/recovery from diagnostics, unlike the KalaNet data to which the Stauch model and Erasmus models were fitted, but does contain detailed information on patients' onset-to-treatment times. A schematic of the model structure, including the multiple compartments for symptomatic infection, is shown in Figure S1. The notation used is as follows: S stands for susceptible, E for latently infected, I for infectious, A for asymptomatically infected, K for symptomatically infected (kala-azar (KA)), T_1 for first-line treatment, T_2 for second-line treatment, R for recovered. Subscripts V denote stages of sandly infection. All model parameters are listed in Table S1 with their definitions and values.

2. Model equations

The system of ordinary differential equations (ODEs) that constitute the transmission model are given below. Given the relatively short time period covered by the data and the unknown relationship between the human and sandfly population sizes, we assume that the human population, N, is constant and the mean sandfly-to-human ratio (SHR) (averaged over seasonal variation), $\overline{n_V(t)} = \overline{N_V(t)/N}$, is constant in the absence of vector control.

The equations for the different human infection stages, in terms of proportions of the total human population, with

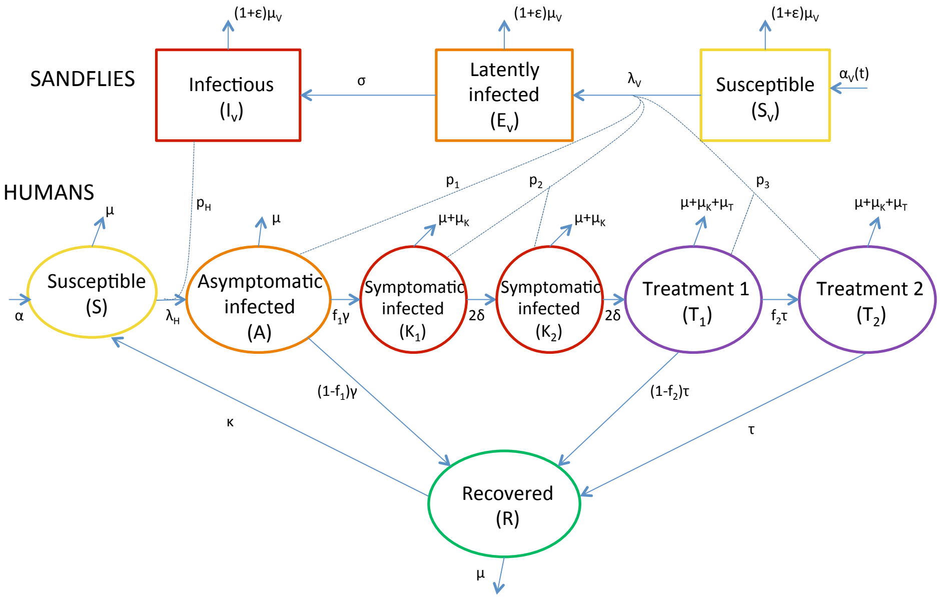

Figure S1. Flow diagram of model structure with 2 sub-compartments for symptomatic infection. Model parameters defined in Table S1 and §2.

2 sub-compartments for symptomatic infection, are:

$$\frac{dS}{dt} = \alpha - (\lambda_H + \mu)S + \kappa R, \tag{1}$$
$$\frac{dA}{dt} = \lambda_H S - (\gamma + \mu)A, \tag{2}$$
$$\frac{dK_1}{dt} = f_1\gamma A - (2\delta + \mu_1)K_1, \tag{3}$$
$$\frac{dK_2}{dt} = 2\delta K_1 - (2\delta + \mu_1)K_2, \tag{4}$$
$$\frac{dT_1}{dt} = 2\delta K_2 - (\tau + \mu_2)T_1, \tag{5}$$
$$\frac{dT_2}{dt} = f_2\tau T_1 - (\tau + \mu_2)T_2, \tag{6}$$
$$\frac{dR}{dt} = (1 - f_1)\gamma A + (1 - f_2)\tau T_1 + \tau T_2 - (\kappa + \mu)R. \tag{7}$$

where all parameters are as defined in Table S1; $\mu_1 = \mu + \mu_K$; $\mu_2 = \mu + \mu_K + \mu_T$; $\alpha = \mu + \mu_K(K_1 + K_2) + (\mu_K + \mu_T)(T_1 + T_2)$ is the birth rate, such that the birth rate balances the net death rate (the natural death rate plus excess mortality due to VL and treatment); and $\lambda_H = \beta p_H n_V I_V$ is the force of infection from sandflies towards humans (assuming frequency-dependent transmission, as is the norm for vector-borne diseases). Following [14], the number of sub-compartments for symptomatic infection for each district is determined by fitting an Erlang distribution (a gamma distribution with positive integer shape parameter $m \in \mathbb{Z}_+$), to the distribution of onset-to-treatment times for that district by maximum likelihood estimation. In other words, a probability density function of the form

$$f(t; m, m\delta) = \frac{(m\delta)^m t^{m-1} \exp(-m\delta t)}{(m-1)!},$$

is fitted to the onset-to-treatment time distribution for each district, where m is the number of sub-compartments and $1/\delta$ is the mean waiting time (so $m\delta$ is the rate parameter for progression to the next sub-compartment). Above the system is given for $m = 2$. With $m = 1$ (1 compartment for symptomatic infection), equations (3)–(5) are replaced by

$$\frac{dK_1}{dt} = f_1\gamma A - (\delta + \mu_1)K_1, \tag{8}$$

$$\frac{dT_1}{dt} = \delta K_1 - (\tau + \mu_2)T_1, \tag{9}$$

and $\alpha = \mu + \mu_K K_1 + (\mu_K + \mu_T)(T_1 + T_2)$. Figure S2 shows the distributions of onset-to-treatment times in 2012 for each district with the fitted Erlang distributions, and Table S3 gives the means and standard deviations of the fitted distributions for 2012 and 2013. The distributions vary considerably between districts, with fitted means and standard deviations ranging from 21.5 days for Begusarai to 63.6 days for W. Champaran in 2012. The best-fit distributions were Erlang distributions with $m = 2$ (2 KA sub-compartments) for Saharsa, E. Champaran, Samastipur, Khagaria, and Patna, and exponential distributions ($m = 1$, i.e. 1 KA compartment) for Gopalganj, Begusarai, and W. Champaran.

Equations (1)–(7) are supplemented by an equation for the cumulative number of VL cases, C,

$$\frac{dC}{dt} = f_1\gamma NA, \tag{10}$$

which is used to fit the model to the data (see below).

In the absence of vector control, the equations for the sandfly infection stages, in terms of proportions of the total sandfly population $N_V(t)$, are:

$$\frac{dS_V}{dt} = \alpha_V(t) - (\lambda_V + \alpha_V(t))S_V, \tag{11}$$

$$\frac{dE_V}{dt} = \lambda_V S_V - (\sigma + \alpha_V(t))E_V, \tag{12}$$

$$\frac{dI_V}{dt} = \sigma E_V - \alpha_V(t)I_V, \tag{13}$$

where $\alpha_V(t) = \mu_V(1 + a_1\cos(\omega t + a_2))$, with $\omega = 2\pi/365$, is the seasonally-varying sandfly birth rate and

$$\lambda_V = \begin{cases} \beta(p_1A + p_2(K_1 + K_2) + p_3(T_1 + T_2)), & \text{for } m = 2 \\ \beta(p_1A + p_2K_1 + p_3(T_1 + T_2)), & \text{for } m = 1 \end{cases} \tag{14}$$

is the force of infection from humans towards sandflies.

The effect of indoor residual spraying of insecticide (IRS) on the sandfly population is modelled via an increase in the sandfly death rate following the start of IRS, from μ_V to $(1 + \epsilon)\mu_V$, where ϵ is proportional to the IRS coverage c with constant of proportionality e (the IRS efficacy factor), i.e. $\epsilon = ec$. The SHR, $n_V(t)$, is therefore given by

$$\frac{dn_V}{dt} = \begin{cases} (\alpha_V(t) - \mu_V)n_V = \mu_V a_1\cos(\omega t + a_2)n_V, & \text{for } t \leq t_{IRS} \\ (\alpha_V(t) - (1+\epsilon)\mu_V)n_V = (-\epsilon\mu_V + \mu_V a_1\cos(\omega t + a_2))n_V, & \text{for } t > t_{IRS}, \end{cases} \tag{15}$$

where t_{IRS} is the start date of IRS. This can be solved analytically to give

$$n_V(t) = \begin{cases} n_V^* \exp\left(\frac{\mu_V a_1}{\omega}\sin(\omega t + a_2)\right), & \text{for } t \leq t_{IRS} \\ n_V^* \exp\left(-\epsilon\mu_V(t - t_{IRS}) + \frac{\mu_V a_1}{\omega}\sin(\omega t + a_2)\right), & \text{for } t > t_{IRS}, \end{cases} \tag{16}$$

where the constant $n_V^* = n_V(0)\exp(-\frac{\mu_V a_1}{\omega}\sin(a_2))$ determines the mean sandfly density. We fit to n_V^* rather than the initial SHR $n_V(0)$, as for a given incidence $n_V(0)$ varies with the phase shift of the seasonal variation in the sandfly birth rate a_2.

Table S1. Model parameters: definitions and values used in model fitting

Parameter	Definition	Value used in model fitting	Source
N	Number of humans	Estimated total population of affected sub-districts in each district on 1st January 2012*	[15]
α	Human birth rate	Set equal to net death rate	-
μ	Human death rate	District-specific (see Table S2)	[16]
$1/\gamma$	Average duration of asymptomatic infection	150 days	[2]
f_1	Proportion of asymptomatic individuals who develop KA	3%	Assumed based on [7]
$1/\delta$	Mean time from onset of KA symptoms to treatment	Erlang distribution fitted to distribution of times for each district (see Table S3)	CARE data
μ_K	Excess mortality rate due to KA	1/150 day^{-1}	Assumed
$1/\tau$	Duration of 1st or 2nd treatment for KA	28 days	CARE data
μ_T	Excess mortality rate due to 1st- or 2nd-line treatment	1/600 day^{-1}	[12, 23]
f_2	Proportion of KA patients who have 2nd treatment	District-specific (see Table S1 in Supplementary File 2 (SF2))	CARE data
$1/\kappa$	Average duration of immunity	5 yrs	Assumed
p_1	Relative infectivity of asymptomatic individuals	0.025	Assumed based on [22]
p_2	Infectivity of individuals with KA	1	Reference value
p_3	Relative infectivity of patients undergoing 1st or 2nd treatment	0.5	Assumed
n_V^*	Baseline effective sandfly-to-human ratio	District-specific	Model fitting
a_1	Relative amplitude of seasonal variation in sandfly birth rate	0.3	[5, 13, 18, 19, 24]
a_2	Phase shift of seasonal variation in sandfly birth rate	$2\pi/3$	[5, 10, 18]
$1/\mu_V$	Average sandfly life expectancy	14 days	[17]
$1/\sigma$	Average duration of latent infection in sandfly	8 days	[9, 17, 20]
β	Sandfly biting rate	1/4 day^{-1}	[8]
c	IRS coverage	District-specific (see Table S1 in SF2)	CARE data
e	IRS efficacy factor	0.006	Parameter uncertainty analysis‡
$\epsilon = ec$	Proportional increase in sandfly death rate due to IRS	District-specific	-
p_H	Probability susceptible human becomes infected when bitten by infectious sandfly	1	Reference value†

* Affected sub-districts are those that have ≥1 KA case during the study period (January 2012–June 2013). Population estimated from annual percentage growth rate between 2001 and 2011.

† p_H is inversely correlated with n_V^*, hence overestimation of p_H is compensated for by underestimation of n_V^* in model fitting.

‡ Average of district maximum likelihood estimates for e in parameter uncertainty analysis excluding W. Champaran, which had a much lower MLE for e than the other districts.

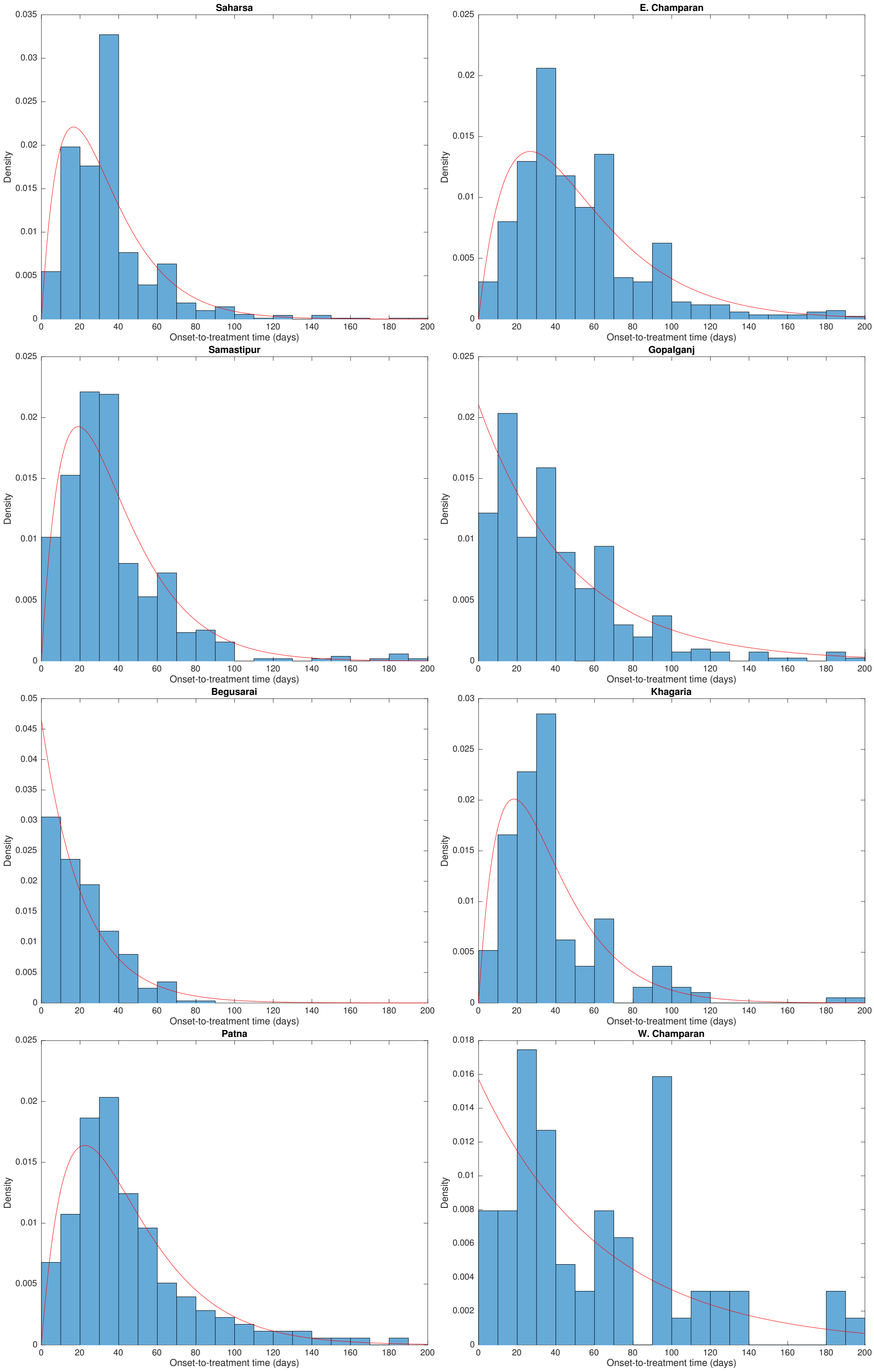

Figure S2. District onset-to-treatment time distributions in 2012 (histograms) and fitted Erlang distributions (red lines). Fitted parameter values for Erlang distributions are given in Table S3.

Table S2. District death rates (from [16]).

District	Death rate (per 1000/yr)
Saharsa	7.6
E. Champaran	7.8
Samastipur	6.7
Gopalganj	6.3
Begusarai	6.2
Khagaria	9.3
Patna	5.0
W. Champaran	8.7

Table S3. Parameter values for fitted onset-to-treatment time (OT) distributions.

District	Number of KA sub-compartments, m	Mean OT, $1/\delta$ (days)		Standard deviation OT, $1/(\delta\sqrt{m})$ (days)	
		2012	2013	2012	2013
Saharsa	2	33.3	34.2	23.5	24.1
E. Champaran	2	53.4	44.1	37.8	31.2
Samastipur	2	38.2	34.1	27.0	24.1
Gopalganj	1	47.5	42.8	47.5	42.8
Begusarai	1	21.5	22.6	21.5	22.6
Khagaria	2	36.6	33.2	25.9	23.5
Patna	2	44.9	43.0	31.7	30.4
W. Champaran	1	63.6	60.6	63.6	60.6

3. Model fitting

The data used for the model fitting consisted of the monthly number of onsets of VL symptoms for each of the 8 study districts from January 2012 to June 2013 (inclusive). The model was fitted separately to the data for each district using maximum likelihood estimation, assuming that the transmission dynamics were in equilibrium prior to 2011 and that IRS started in 2011. The number of VL onsets in month T, M_T, was assumed to be Poisson distributed with rate parameter $\eta_T = C_T - C_{T-1}$, where C_T is the cumulative number of VL onsets at the end of month T predicted by the model, i.e.

$$\mathbb{P}(M_T = m_T) = \frac{\eta_T^{m_T}\exp(-\eta_T)}{m_T!}. \tag{17}$$

The full likelihood for each district was taken as the product of the probabilities of the 18 monthly case numbers $\{m_T\}_{T=1,\ldots,18}$

$$L = \prod_{T=1}^{18}\mathbb{P}(M_T = m_T) = \prod_{T=1}^{18}\frac{\eta_T^{m_T}}{m_T!}\exp\left(-\sum_{T=1}^{18}\eta_T\right). \tag{18}$$

The baseline SHR, n_V^*, was estimated for each district by maximising the log-likelihood

$$\log L = \sum_{T=1}^{18}(m_T\log\eta_T - \eta_T - \log(m_T!)), \tag{19}$$

using the `interior-point` algorithm in the `fmincon` function in `MATLAB` (version 8.6). For each evaluation of the likelihood, the model was run for 200 years prior to the start of the data in 2012 so that the transmission dynamics reached equilibirum.

3.1. Calculating R_0

A key parameter in the transmission dynamics of an infectious disease is the basic reproduction number, R_0, defined for a homogeneously mixing population as the average number of secondary cases caused by a single infectious case when the population is entirely susceptible and there are no interventions. If $R_0 > 1$, the disease can spread through the population and a stable endemic equilibrium can be established. If $R_0 \leq 1$, the only equilibrium is the stable disease-free equilibrium and the disease will eventually die out.

Since we assume in the model that the population is homogeneously mixed (which is a limitation of the model, see Discussion in main text), we require $R_0 > 1$ for there to be an endemic equilibrium. However, the incidence of VL at the district-level is very low, so the range of parameter values for which $R_0 > 1$ and the model gives a good fit to the district incidences is very small. Thus, for each set of parameter values proposed in the fitting algorithm we calculate R_0 before solving the ODE system (1)–(14) to avoid wasted calculations when $R_0 \leq 1$.

3.1.1. Without seasonality in sandfly population

For vector-borne diseases, such as VL, R_0 depends on the vector-to-host ratio, and a common simplifying assumption is to treat the vector-to-host ratio as constant throughout the year. If this assumption were appropriate for the Indian subcontinent, i.e. if $n_V = N_V/N \approx$ const., we could calculate R_0 for the system of equations (1)–(14) by the standard next generation matrix approach [4] to obtain

$$R_0 = \sqrt{\frac{N_V \beta^2 p_H}{N \mu_V} \frac{\sigma}{\sigma + \mu_V} \frac{1}{\gamma + \mu} \left[p_1 + f_1 \gamma D_K \left(p_2 + 2\delta D_K \left(p_2 + 2\delta D_T p_3 \left(1 + f_2 \tau D_T\right)\right)\right)\right]}, \tag{20}$$

for 2 KA sub-compartments, where $D_K = 1/(2\delta + \mu + \mu_K)$ and $D_T = 1/(\tau + \mu + \mu_K + \mu_T)$ are the mean durations of the symptomatic stages and first/second treatment (accounting for mortality). From this expression we can see that, in the absence of seasonality, the SHR N_V/N and the asymptomatic infectivity p_1 cannot be individually estimated just from the incidence of symptomatic infection (which determines R_0). This expression also suggests that the proportion of asymptomatic individuals progressing to clinical VL, f_1, and the average duration of asymptomatic infection, $1/\gamma$, are likely to be strongly correlated for a given incidence, so that they cannot be estimated separately from the data; and that the relative infectivity of individuals under first/second treatment, p_3, is also likely to be unidentifiable.

3.1.2. With seasonal variation in sandfly population

The SHR in India is clearly not constant throughout the year, however, as sandflies show marked seasonal variation in numbers (see references in Table S1 and main text). To account for this it is necessary to extend the definition of R_0 to cater for periodic variation in the sandfly population. We calculate R_0 for our model following the approach of Bacaër [1], which is an extension of the next generation matrix method.

First we reduce the system (1)–(14) to equations for just the set of infected states $\{A, K_1, K_2, T_1, T_2, E_V, I_V\}$ ((2)–(6) and (12)–(13)), known as the infection subsystem. Then we linearise the infection subsystem about the disease-free equilibrium ($S = 1$, $S_V = 1$, $A, K_1, K_2, T_1, T_2, E_V, I_V = 0$), and write down matrices of the transmissions, T, and

transitions, Σ, associated with the infected states:

$$T = \begin{pmatrix} 0 & \dots & \dots & \dots & 0 & 0 & \beta p_H n_V(t) \\ \vdots & \ddots & & & \vdots & \vdots & 0 \\ \vdots & & \ddots & & \vdots & \vdots & \vdots \\ \vdots & & & \ddots & \vdots & \vdots & \vdots \\ 0 & \dots & \dots & \dots & 0 & 0 & 0 \\ \beta p_1 & \beta p_2 & \beta p_2 & \beta p_3 & \beta p_3 & 0 & 0 \\ 0 & \dots & \dots & \dots & \dots & 0 & 0 \end{pmatrix}, \tag{21}$$

$$\Sigma = \begin{pmatrix} \gamma+\mu & 0 & \dots & \dots & \dots & \dots & 0 \\ -f_1\gamma & 2\delta+\mu_1 & \ddots & & & & \vdots \\ 0 & -2\delta & 2\delta+\mu_1 & \ddots & & & \vdots \\ \vdots & \ddots & -2\delta & \tau+\mu_2 & \ddots & & \vdots \\ 0 & \dots & 0 & -f_2\tau & \tau+\mu_2 & \ddots & \vdots \\ 0 & \dots & \dots & \dots & 0 & \sigma+\alpha_V(t) & 0 \\ 0 & \dots & \dots & \dots & 0 & \sigma & \alpha_V(t) \end{pmatrix} \tag{22}$$

With these definitions we can write the linearised infection subsystem as

$$\frac{d\mathbf{x}}{dt} = (T(t) - \Sigma(t))\mathbf{x}(t), \tag{23}$$

where $\mathbf{x} = (A, K_1, K_2, T_1, T_2, E_V, I_V)^T$, and T and Σ are matrix-valued periodic functions of t with period $p = 365$ days. To determine R_0 we need to determine the condition for this system to have a non-trivial stable p-periodic solution (i.e. for there to be a stable endemic equilibrium). It transpires that this condition is equivalent to R_0 being the unique positive root of the equation

$$\rho(X(p, \lambda)) = 1, \tag{24}$$

where ρ is the spectral radius (the largest eigenvalue in absolute value) of the fundamental matrix solution $X(t, \lambda)$ at time $t = p$ of

$$\frac{\partial}{\partial t}X(t,\lambda) = \left(\frac{T(t)}{\lambda} - \Sigma(t)\right)X(t,\lambda), \tag{25}$$

$$X(0) = I_7, \tag{26}$$

where I_7 is the 7×7 identity matrix (see [1, 4] for details). We therefore calculate R_0 by combining a root-finding algorithm (the `trust-region-dogleg` algorithm under the `fsolve` function in `MATLAB`) with a numerical method for solving (25)–(26). For each value of λ, $X(p, \lambda)$ is calculated by solving (25)–(26) over one period with the 7 standard unit vectors of $\mathbb{R}^7$ as initial conditions (using the `MATLAB` solver `ode45`) and taking the resulting vectors at $t = p$ as the columns of X.

4. Parameter uncertainty analysis

Due to the lack of data available to accurately quantify many parts of the transmission process, e.g. the durations of different stages of infection and the infectivity of individuals in these stages to sandflies, there is a large amount of uncertainty in the values of many of the model parameters. We therefore conducted a parameter uncertainty analysis to determine the ranges of parameter values for which the model gives a good fit to the data. Nine parameters were considered: the baseline SHR, n_V^*; the amplitude and phase shift of the seasonal variation in the sandfly birth rate, a_1

and a_2; the proportion of asymptomatic individuals who develop clinical VL, f_1; the average duration of asymptomatic infection, $1/\gamma$; the relative infectivities of asymptomatic individuals and individuals undergoing treatment, p_1 and p_3; the duration of immunity, $1/\kappa$; and the IRS efficacy factor e. Ninety-five per cent confidence intervals for these parameters were determined by sampling 300,000 parameter sets from bounded uniform distributions, simulating the model with these parameter sets and accepting those for which the goodness of fit was close to that for the maximum likelihood estimates (MLEs) using the likelihood ratio test (i.e. those for which the log-likelihood differed by less than $\chi_1^2(0.95)/2 = 1.92$ from the maximum log-likelihood, $(\log L_{MLE} - \log L) < \chi_1^2(0.95)/2)$. The upper bound for the proportion of asymptomatic individuals who develop clinical VL was chosen based on an assumed asymptomatic infection prevalence of 1% at district level [3], the largest estimated district incidence being approximately 5/10,000/yr and the longest estimate for the average asymptomatic infection duration in [11] being roughly 530 days, so that, assuming the transmission dynamics are in equilibrium,

$$f_{1,\max}\gamma \approx \frac{[\text{max. VL incidence}]}{A_{\text{eqm}}} = \frac{0.0005}{0.01} = 0.05/\text{yr}$$

$$\Rightarrow f_{1,\max} = \frac{0.05}{\gamma} = \frac{0.05}{365/530} = 0.073.$$

The lower bound for f_1 was chosen as 1% since the lowest estimate from field studies is 3.48% [6] and a value lower than 1% therefore seems implausible. The lower and upper bounds for the other parameters were chosen to give relatively broad ranges around the fixed values used in the analysis in the main text and based on plausible bounds from parameter estimates in the literature. The proposed and accepted ranges for all the parameter values and the maximum likelihood parameter estimates for each district are shown in Table S4.

The confidence intervals (CIs) for most of the parameters are wide across all the districts indicating that the data is equally well explained by a large range of parameter values. However, the accepted ranges for the phase shift in the seasonal forcing of the sandfly birth rate are clearly tighter than the proposed range, apart from for the two least endemic districts (Patna and West Champaran), covering 1.83–2.72 (corresponding to a peak in the sandfly population between late October and late November). The maximum likelihood parameter estimates also vary considerably across districts. The MLEs for the duration of asymptomatic infection range between 98 and 163 days, but are mostly in the range 110-150 days, which agrees with our previous estimate of 147 days (95% CI 130–166 days) [2], and together with the peaks in clinical cases in January–April suggests a peak in transmission in August–November (in the preceding year), coinciding with the seasonal peak in sandfly density.

Figure S3 shows the parameter values for each district for which the model likelihood is close to the maximum likelihood, specifically, for which the difference in the log-likelihood is less than $\chi_9^2(0.95)/2 = 8.46$, i.e. not significant by the likelihood ratio test with 9 degrees of freedom at significance level 0.05 (red dots); and parameter values for which the likelihood is significantly different (blue dots). Below Figure S3 are matrices of the parameter correlation coefficients (defined for vectors of parameter values X and Y as $\text{cov}(X,Y)/\sqrt{\text{var}(X)\text{var}(Y)}$, where $\text{cov}(X,Y)$ is the covariance of X and Y and $\text{var}(X)$ is the variance of X, so that 1 represents perfect positive correlation, -1 perfect negative correlation and 0 no correlation) for the high-likelihood parameter sets (red dots in Figure S3) for each district. It is clear from the plots and matrices that the baseline SHR n_V^* and the relative infectivity of asymptomatic individuals p_1 are strongly negatively correlated (with correlation coefficients ranging between -0.89 and -0.56 for the different districts), as might be expected from the expression for R_0 without seasonality (20). The amplitude of the seasonal forcing of the sandfly birth rate a_1 and the mean duration of asymptomatic infection $1/\gamma$ are positively correlated for most districts. This is because a longer asymptomatic duration (larger $1/\gamma$) dampens the seasonality in VL incidence caused by seasonality in the sandfly birth rate, which can be compensated for by a greater amplitude in the seasonal variation in the birth rate (greater a_1). The IRS efficacy e and $1/\gamma$ are also positively correlated for most districts, as a greater reduction in sandfly density due to a higher IRS efficacy can counterbalance more transmission from asymptomatic individuals due to a longer asymptomatic duration to give similar VL incidence. These observations imply that n_V^*, p_1, a_1, $1/\gamma$ and e cannot be uniquely determined from the data. Thus the district-level estimates for n_V^* in the main paper should be viewed as relative estimates, as they are only unique up to the choice of values for p_1 and a_1 (in addition to being underestimates due to choosing $p_H = 1$). The wide CIs and even distribution of points in the scatter plots for the mean duration of immunity, $1/\kappa$, show that there is insufficient information in the data to estimate this parameter with any precision. In the simulations in the main text we therefore assume a value of 5 years for $1/\kappa$, and discuss issues related to the unknown duration of immunity in the Discussion.

Figure S4 shows the predicted VL incidence for each district with the parameter MLEs and continuation of existing interventions. Table S5 gives the MLE and 95% CI for the elimination month for each district, defined as the month in which the incidence decreases below 1/10,000/yr and thereafter stays below this level. Confidence intervals were determined by taking the minimum and maximum predicted incidence at each time point across the model outputs for all the accepted parameter sets. The MLEs for the elimination month span from April 2013 for Begusarai to March 2016 for Saharsa, and agree well with those from the model fitting in the main text. All the confidence intervals for the elimination month span 1–2 years, except those for E. Champaran (April 2013–May 2013) and Khagaria (March 2014–March 2018). The narrow confidence interval for E. Champaran likely reflects the strong seasonal pattern in its monthly numbers of VL cases (see Figure S1 in Supplementary File 2), which can only be reproduced by small ranges of parameter values. Conversely, the very wide confidence interval for Khagaria reflects the lack of a clear seasonal pattern in its VL case numbers, which can be generated by large ranges of parameter values.

Table S4. Parameter uncertainty analysis: proposed and accepted ranges for parameter values and maximum likelihood estimates for each district. Sh = Saharsa, EC = East Champaran, Sm = Samastipur, G = Gopalganj, B = Begusarai, K = Khagaria, P = Patna, WC = West Champaran.

Parameter	Proposed range*	Accepted range†								Maximum likelihood estimate							
		Sh	EC	Sm	G	B	K	P	WC	Sh	EC	Sm	G	B	K	P	WC
Sandfly-to-human ratio, n_V^*	0.1–0.6	0.14–0.59	0.11–0.54	0.31–0.47	0.14–0.46	0.10–0.59	0.10–0.60	0.10–0.59	0.13–0.60	0.34	0.27	0.46	0.35	0.59	0.17	0.36	0.35
Seasonal forcing amplitude, a_1	0.1–0.5	0.19–0.50	0.32–0.50	0.31–0.35	0.29–0.45	0.15–0.49	0.23–0.46	0.10–0.47	0.10–0.33	0.29	0.37	0.34	0.34	0.24	0.40	0.12	0.14
Seasonal phase shift, a_2	$\frac{7\pi}{12}$–π	1.90–2.28	2.27–2.65	1.87–1.91	1.84–1.95	1.83–2.39	2.07–2.72	1.83–3.05	1.85–2.98	2.11	2.42	1.88	1.88	1.97	2.43	2.33	2.52
Fraction of asymptomatic individuals who develop VL, f_1	0.01–0.073	0.010–0.073	0.010–0.063	0.022–0.063	0.013–0.063	0.011–0.068	0.010–0.071	0.010–0.072	0.010–0.063	0.013	0.019	0.022	0.027	0.028	0.020	0.013	0.043
Average duration of asymptomatic infection, $1/\gamma$ (days)	90–180	115–180	98–153	97–118	92–169	95–177	102–176	92–179	93–177	163	109	116	117	98	141	123	122
Relative infectivity of asymptomatic individuals, p_1	0.01–0.07	0.013–0.067	0.015–0.065	0.015–0.020	0.017–0.049	0.011–0.068	0.012–0.068	0.010–0.070	0.011–0.070	0.030	0.035	0.015	0.021	0.016	0.044	0.034	0.018
Relative infectivity of patients under treatments 1 and 2, p_3	0.1–1	0.14–1.00	0.12–0.97	0.61–0.91	0.26–0.97	0.10–1.00	0.14–0.96	0.10–0.99	0.14–1.00	0.58	0.84	0.91	0.83	0.98	0.22	0.64	0.95
Average duration of immunity, $1/\kappa$ (yrs)	3–8	3.5–8.0	3.2–7.9	4.4–6.5	3.2–7.9	3.3–8.0	3.0–7.9	3.0–8.0	3.1–7.9	4.5	6.8	6.4	5.5	5.1	4.2	4.1	6.8
IRS efficacy factor, e	0–0.015	0.003–0.008	0.008–0.014	0.003–0.004	0.003–0.008	0.003–0.010	0.001–0.006	0.003–0.015	0.0001–0.007	0.005	0.008	0.003	0.005	0.005	0.003	0.010	0.0004

* Simulations run with 300,000 parameter sets sampled from random uniform distributions over proposed parameter ranges

† Parameter sets accepted if model log-likelihood (19) was within $\chi_1^2(0.95)/2 = 1.92$ of maximum log-likelihood over all simulations.

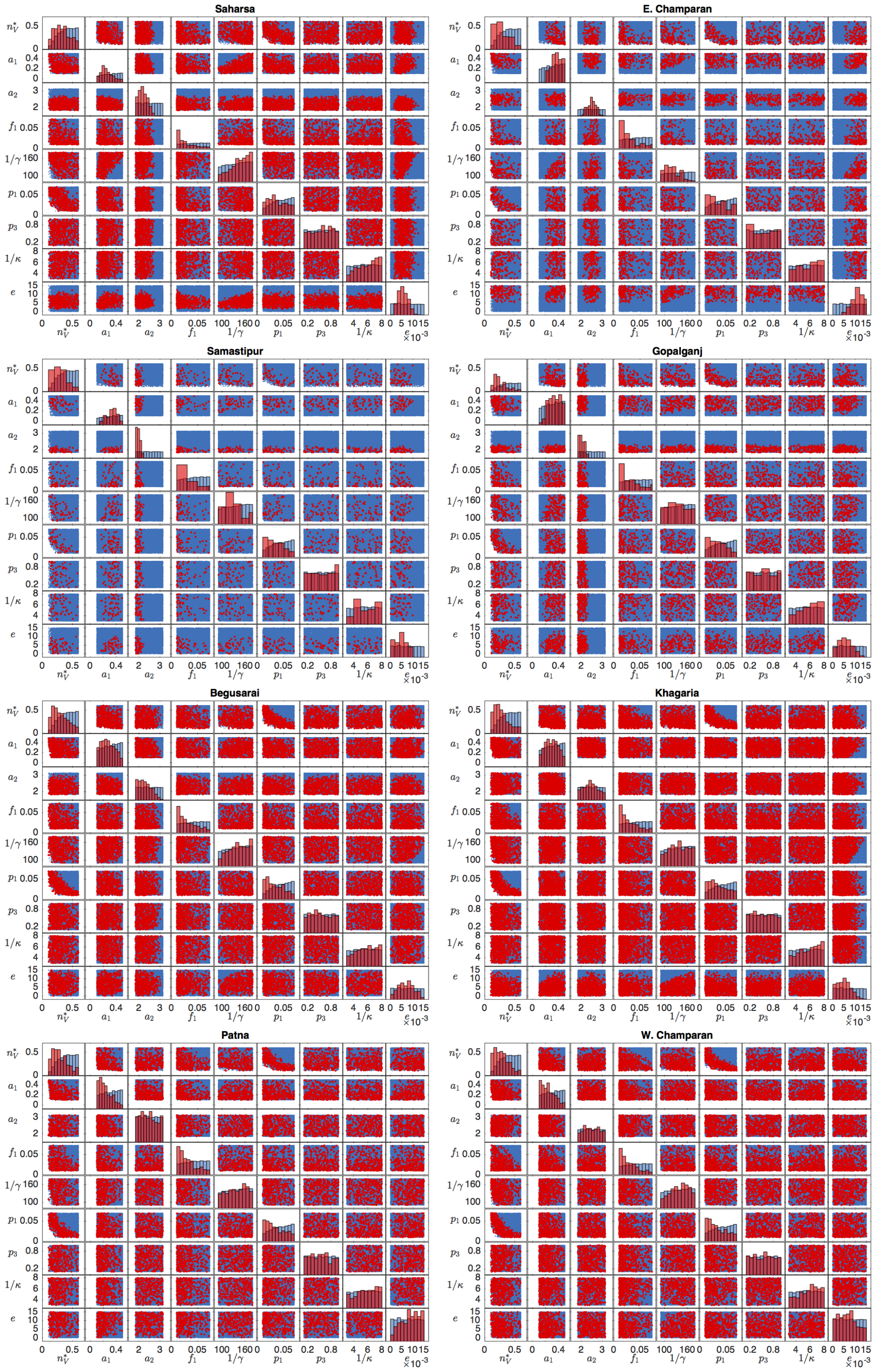

Figure S3. Parameter uncertainty analysis: scatter matrix plots of the proposed parameter values for each district for which $R_0 > 1$ (blue dots) and the parameter sets for which the model log-likelihood was within $\chi^2_9(0.95)/2 = 8.46$ of the maximum log-likelihood (red dots). Marginal distributions of proposed parameters (blue histograms) and parameters for which the model likelihood was not significantly different from the maximum likelihood (red histograms) shown on the main diagonal. Parameters considered: baseline sandfly-to-human ratio n_V^*, amplitude and phase shift of seasonal forcing in sandfly birth rate a_1 and a_2, proportion of asymptomatic individuals who develop clinical VL f_1, mean duration of asymptomatic infection $1/\gamma$, relative infectivities of asymptomatic individuals and individuals undergoing treatment p_1 and p_3, mean duration of immunity $1/\kappa$, and IRS efficacy factor e. Each blue dot represents one of 10,000 simulations (300,000 simulations were run for each district). Matrices of the parameter correlation coefficients for each district are presented below.

Saharsa:

	n_V^*	a_1	a_2	f_1	$1/\gamma$	p_1	p_3	$1/\kappa$	e
n_V^*	1.00	-0.27	0.03	-0.25	-0.19	-0.56	0.04	0.07	0.01
a_1	-0.27	1.00	-0.24	-0.11	0.50	-0.27	-0.02	-0.14	0.32
a_2	0.03	-0.24	1.00	0.22	-0.22	0.16	0.02	0.29	0.04
f_1	-0.25	-0.11	0.22	1.00	-0.35	-0.01	-0.11	0.38	-0.10
$1/\gamma$	-0.19	0.50	-0.22	-0.35	1.00	-0.35	0.02	-0.32	0.31
p_1	-0.56	-0.27	0.16	-0.01	-0.35	1.00	-0.04	0.24	-0.02
p_3	0.04	-0.02	0.02	-0.11	0.02	-0.04	1.00	-0.01	-0.01
$1/\kappa$	0.07	-0.14	0.29	0.38	-0.32	0.24	-0.01	1.00	0.09
e	0.01	0.32	0.04	-0.10	0.31	-0.02	-0.01	0.09	1.00

E. Champaran:

	n_V^*	a_1	a_2	f_1	$1/\gamma$	p_1	p_3	$1/\kappa$	e
n_V^*	1.00	-0.34	-0.11	-0.32	-0.21	-0.70	0.00	0.07	-0.02
a_1	-0.34	1.00	-0.06	-0.02	0.45	-0.14	-0.02	0.07	0.25
a_2	-0.11	-0.06	1.00	0.29	0.26	0.03	-0.36	-0.15	0.27
f_1	-0.32	-0.02	0.29	1.00	0.17	-0.02	-0.34	-0.25	0.39
$1/\gamma$	-0.21	0.45	0.26	0.17	1.00	-0.13	-0.39	-0.29	0.61
p_1	-0.70	-0.14	0.03	-0.02	-0.13	1.00	-0.03	-0.10	-0.20
p_3	0.00	-0.02	-0.36	-0.34	-0.39	-0.03	1.00	0.37	-0.47
$1/\kappa$	0.07	0.07	-0.15	-0.25	-0.29	-0.10	0.37	1.00	-0.39
e	-0.02	0.25	0.27	0.39	0.61	-0.20	-0.47	-0.39	1.00

Samastipur:

	n_V^*	a_1	a_2	f_1	$1/\gamma$	p_1	p_3	$1/\kappa$	e
n_V^*	1.00	-0.12	-0.51	-0.24	-0.53	-0.89	0.54	0.30	-0.41
a_1	-0.12	1.00	-0.01	-0.02	0.23	-0.16	0.15	0.10	0.18
a_2	-0.51	-0.01	1.00	-0.09	0.30	0.51	-0.49	-0.39	0.21
f_1	-0.24	-0.02	-0.09	1.00	-0.06	0.02	-0.27	-0.13	-0.14
$1/\gamma$	-0.53	0.23	0.30	-0.06	1.00	0.29	-0.07	-0.16	0.60
p_1	-0.89	-0.16	0.51	0.02	0.29	1.00	-0.52	-0.31	0.30
p_3	0.54	0.15	-0.49	-0.27	-0.07	-0.52	1.00	0.32	-0.14
$1/\kappa$	0.30	0.10	-0.39	-0.13	-0.16	-0.31	0.32	1.00	-0.02
e	-0.41	0.18	0.21	-0.14	0.60	0.30	-0.14	-0.02	1.00

Gopalganj:

	n_V^*	a_1	a_2	f_1	$1/\gamma$	p_1	p_3	$1/\kappa$	e
n_V^*	1.00	-0.28	-0.29	-0.37	-0.44	-0.76	0.33	-0.18	-0.13
a_1	-0.28	1.00	0.03	-0.04	0.39	-0.15	-0.02	0.12	0.28
a_2	-0.29	0.03	1.00	0.07	0.35	0.33	-0.49	0.19	0.10
f_1	-0.37	-0.04	0.07	1.00	-0.05	0.11	-0.15	0.16	0.02
$1/\gamma$	-0.44	0.39	0.35	-0.05	1.00	0.11	-0.22	0.18	0.20
p_1	-0.76	-0.15	0.33	0.11	0.11	1.00	-0.41	0.12	0.06
p_3	0.33	-0.02	-0.49	-0.15	-0.22	-0.41	1.00	-0.14	-0.22
$1/\kappa$	-0.18	0.12	0.19	0.16	0.18	0.12	-0.14	1.00	0.17
e	-0.13	0.28	0.10	0.02	0.20	0.06	-0.22	0.17	1.00

Begusarai:

	n_V^*	a_1	a_2	f_1	$1/\gamma$	p_1	p_3	$1/\kappa$	e
n_V^*	1.00	-0.32	-0.46	-0.18	-0.71	-0.83	0.63	-0.30	-0.39
a_1	-0.32	1.00	0.02	-0.02	0.25	0.01	-0.14	0.04	0.18
a_2	-0.46	0.02	1.00	0.08	0.46	0.33	-0.41	0.17	0.16
f_1	-0.18	-0.02	0.08	1.00	0.08	0.01	-0.09	0.06	0.02
$1/\gamma$	-0.71	0.25	0.46	0.08	1.00	0.38	-0.59	0.23	0.47
p_1	-0.83	0.01	0.33	0.01	0.38	1.00	-0.50	0.26	0.24
p_3	0.63	-0.14	-0.41	-0.09	-0.59	-0.50	1.00	-0.24	-0.34
$1/\kappa$	-0.30	0.04	0.17	0.06	0.23	0.26	-0.24	1.00	0.18
e	-0.39	0.18	0.16	0.02	0.47	0.24	-0.34	0.18	1.00

Khagaria:

	n_V^*	a_1	a_2	f_1	$1/\gamma$	p_1	p_3	$1/\kappa$	e
n_V^*	1.00	-0.45	0.00	-0.03	-0.19	-0.76	0.28	0.30	0.19
a_1	-0.45	1.00	-0.11	-0.25	0.09	0.07	-0.37	-0.34	-0.10
a_2	0.00	-0.11	1.00	-0.02	0.02	0.08	-0.04	0.04	-0.03
f_1	-0.03	-0.25	-0.02	1.00	-0.06	-0.16	0.20	0.23	0.18
$1/\gamma$	-0.19	0.09	0.02	-0.06	1.00	-0.12	-0.03	-0.08	0.18
p_1	-0.76	0.07	0.08	-0.16	-0.12	1.00	-0.20	-0.15	-0.24
p_3	0.28	-0.37	-0.04	0.20	-0.03	-0.20	1.00	0.39	0.34
$1/\kappa$	0.30	-0.34	0.04	0.23	-0.08	-0.15	0.39	1.00	0.27
e	0.19	-0.10	-0.03	0.18	0.18	-0.24	0.34	0.27	1.00

Patna:

	n_V^*	a_1	a_2	f_1	$1/\gamma$	p_1	p_3	$1/\kappa$	e
n_V^*	1.00	-0.40	-0.01	-0.43	-0.31	-0.71	0.12	-0.26	-0.00
a_1	-0.40	1.00	0.02	0.31	0.38	-0.08	-0.20	0.42	-0.02
a_2	-0.01	0.02	1.00	0.19	0.14	-0.09	-0.12	0.19	-0.10
f_1	-0.43	0.31	0.19	1.00	0.18	-0.00	-0.15	0.39	-0.09
$1/\gamma$	-0.31	0.38	0.14	0.18	1.00	-0.14	-0.12	0.27	-0.03
p_1	-0.71	-0.08	-0.09	-0.00	-0.14	1.00	-0.08	0.02	-0.03
p_3	0.12	-0.20	-0.12	-0.15	-0.12	-0.08	1.00	-0.17	0.10
$1/\kappa$	-0.26	0.42	0.19	0.39	0.27	0.02	-0.17	1.00	-0.11
e	-0.00	-0.02	-0.10	-0.09	-0.03	-0.03	0.10	-0.11	1.00

W. Champaran:

	n_V^*	a_1	a_2	f_1	$1/\gamma$	p_1	p_3	$1/\kappa$	e
n_V^*	1.00	-0.31	0.08	-0.13	-0.26	-0.79	0.18	0.10	-0.29
a_1	-0.31	1.00	-0.07	-0.31	0.22	0.10	-0.36	-0.25	0.33
a_2	0.08	-0.07	1.00	0.07	0.00	-0.10	0.09	0.09	-0.08
f_1	-0.13	-0.31	0.07	1.00	-0.26	-0.16	0.28	0.21	-0.20
$1/\gamma$	-0.26	0.22	0.00	-0.26	1.00	0.12	-0.28	-0.17	0.39
p_1	-0.79	0.10	-0.10	-0.16	0.12	1.00	-0.28	-0.11	0.32
p_3	0.18	-0.36	0.09	0.28	-0.28	-0.28	1.00	0.24	-0.42
$1/\kappa$	0.10	-0.25	0.09	0.21	-0.17	-0.11	0.24	1.00	-0.22
e	-0.29	0.33	-0.08	-0.20	0.39	0.32	-0.42	-0.22	1.00

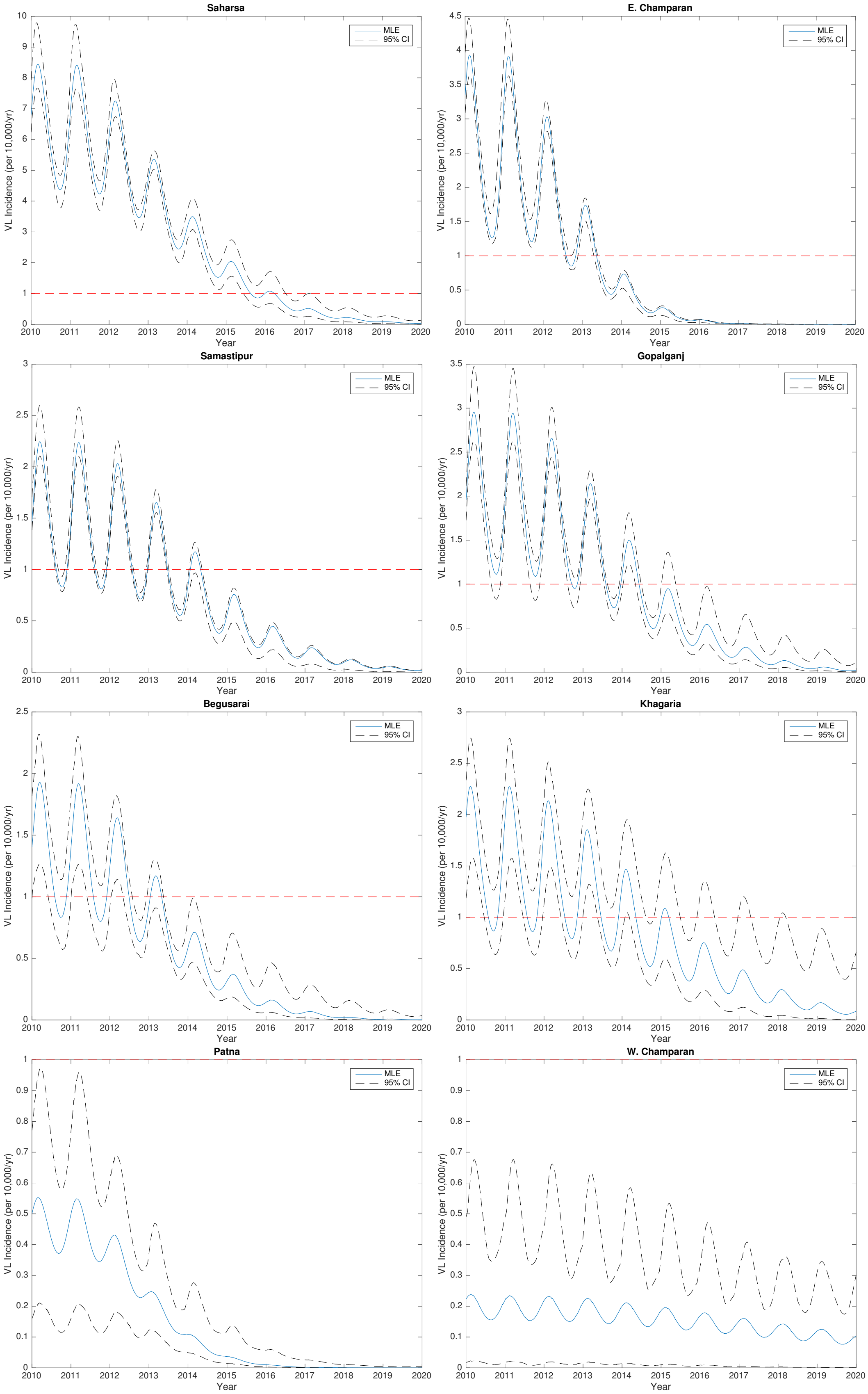

Figure S4. Maximum likelihood estimates (MLE) and 95% confidence intervals (CIs) for predicted VL incidence for each district up to 2020 from parameter uncertainty analysis

Table S5. Maximum likelihood estimates (MLEs) of elimination month for each district with 95% confidence intervals (CI) and corresponding negative log-likelihoods. Elimination month defined as the month in which incidence decreased below 1/10,000/yr and then remained below this level.

District	$\min(-\log L)$	MLE for elimination month	95% CI for elimination month
Saharsa	72.3	Mar 2016	Jun 2015 - Jul 2016
E. Champaran	82.3	May 2013	Apr 2013 - May 2013
Samastipur	67.6	Apr 2014	Jun 2013 - May 2014
Gopalganj	59.9	Jun 2014	May 2014 - May 2015
Begusarai	53.6	Apr 2013	May 2012 - May 2013
Khagaria	64.5	Mar 2015	Mar 2014 - Mar 2018
Patna	47.8	*	*
W. Champaran	42.2	*	*

* Incidence was already <1/10,000/yr in these districts in 2012.

References

[1] Bacaër, N., 2007. Approximation of the basic reproduction number R0 for vector-borne diseases with a periodic vector population. Bulletin of Mathematical Biology 69 (3), 1067–1091. doi:10.1007/s11538-006-9166-9.

[2] Chapman, L. A., Dyson, L., Courtenay, O., Chowdhury, R., Bern, C., Medley, G. F., Hollingsworth, T. D., 2015. Quantification of the natural history of visceral leishmaniasis and consequences for control. Parasites & Vectors 8 (1), 1. doi:10.1186/s13071-015-1136-3.

[3] Das, V. N. R., Pandey, R. N., Siddiqui, N. A., Chapman, L. A., Kumar, V., Pandey, K., Matlashewski, G., Das, P., 2016. Longitudinal Study of Transmission in Households with Visceral Leishmaniasis, Asymptomatic Infections and PKDL in Highly Endemic Villages in Bihar, India. PLOS Neglected Tropical Diseases 10 (12), e0005196. doi:10.1371/journal.pntd.0005196.

[4] Diekmann, O., Heesterbeek, H., Britton, T., 2012. Mathematical tools for understanding infectious disease dynamics. Princeton University Press.

[5] Ghosh, K., Mukhopadhyay, J., Desai, M. M., Senroy, S., Bhattacharya, A., 1999. Population ecology of Phlebotomus argentipes (Diptera: Psychodidae) in West Bengal, India. Journal of Medical Entomology 36 (5), 588–594.

[6] Gidwani, K., Rai, M., Chakravarty, J., Boelaert, M., Sundar, S., 2009. Short Report: Evaluation of leishmanin skin test in Indian visceral leishmaniasis. American Journal of Tropical Medicine and Hygiene 80 (4), 566–567.

[7] Hasker, E., Malaviya, P., Gidwani, K., Picado, A., Ostyn, B., Kansal, S., Singh, R. P., Singh, O. P., Chourasia, A., Kumar Singh, A., Shankar, R., Wilson, M. E., Khanal, B., Rijal, S., Boelaert, M., Sundar, S., 2014. Strong Association between Serological Status and Probability of Progression to Clinical Visceral Leishmaniasis in Prospective Cohort Studies in India and Nepal. PLoS Neglected Tropical Diseases 8 (1), 19. doi:10.1371/journal.pntd.0002657.

[8] Hati, A., Palit, A., Chakraborty, S., Bhattacharya, S., Ghosh, K., Das, S., 1984. Phlebotomus argentipes annadale and brunetti (diptera) caught on man baits at night in clean biotope. Records of the Zoological Survey of India 81, 9–12.

[9] Hurwitz, I., Hillesland, H., Fieck, A., Das, P., Durvasula, R., 2011. The paratransgenic sand fly: A platform for control of leishmania transmission. Parasites & Vectors 4 (1), 1–9. doi:10.1186/1756-3305-4-82.

[10] Kesari, S., Mandal, R., Bhunia, G. S., Kumar, V., Das, P., 2014. Spatial distribution of P. argentipes in association with agricultural surrounding environment in North Bihar, India. Journal of Infection in Developing Countries 8 (3), 358–64. doi:10.3855/jidc.3353.

[11] Le Rutte, E. A., Coffeng, L. E., Bontje, D. M., Hasker, E. C., Postigo, J. A. R., Argaw, D., Boelaert, M. C., De Vlas, S. J., 2016. Feasibility of eliminating visceral leishmaniasis from the Indian subcontinent: explorations with a set of deterministic age-structured transmission models. Parasites & Vectors 9 (1), 1. doi:10.1186/s13071-016-1292-0.

[12] Maintz, E.-M., Hassan, M., Huda, M. M., Ghosh, D., Hossain, M. S., Alim, A., Kroeger, A., Arana, B., Mondal, D., 2014. Introducing single dose liposomal amphotericin B for the treatment of visceral leishmaniasis in rural bangladesh: feasibility and acceptance to patients and health staff. Journal of tropical medicine 2014.

[13] Malaviya, P., Hasker, E., Picado, A., Mishra, M., Van Geertruyden, J.-P., Das, M. L., Boelaert, M., Sundar, S., 2014. Exposure to Phlebotomus argentipes (Diptera, Psychodidae, Phlebotominae) sand flies in rural areas of Bihar, India: the role of housing conditions. PLoS One 9 (9), e106771. doi:10.1371/journal.pone.0106771.

[14] Mubayi, A., Castillo-Chavez, C., Chowell, G., Kribs-Zaleta, C., Ali Siddiqui, N., Kumar, N., Das, P., 2010. Transmission dynamics and underreporting of Kala-azar in the Indian state of Bihar. Journal of Theoretical Biology 262 (1), 177–185. doi:10.1016/j.jtbi.2009.09.012.

[15] Office of the Registrar General & Census Commissioner, India, Ministry of Home Affairs, Government of India, 2011. Census Of India, 2011. http://www.census2011.co.in/census/state/districtlist/bihar.html, (accessed 4/1/17).

[16] Office of the Registrar General & Census Commissioner, India, Ministry of Home Affairs, Government of India, 2012. Bihar Annual Health Survey Bulletin 2011-12. http://www.censusindia.gov.in/vital_statistics/AHSBulletins/files2012/Bihar_Bulletin%202011-12.pdf, (accessed 4/1/17).

[17] Palit, A., Bhattacharya, S. K., Kundu, S., 2010. Gonotrophic cycle and age gradation of Phlebotomus argentipes in West Bengal, India. In: 22nd National Congress of Parasitology, Oct. 30-Nov. 01, 2011. Dept. of Zoology, University of Kalyani, Kalyani, West Bengal, pp. 10–17.

[18] Picado, A., Das, M. L., Kumar, V., Dinesh, D. S., Rijal, S., Singh, S. P., Das, P., Coosemans, M., Boelaert, M., Davies, C., 2010. Phlebotomus argentipes seasonal patterns in India and Nepal. Journal of medical entomology 47 (2), 283–286. doi:10.1603/ME09175.

[19] Poché, D., Garlapati, R., Ingenloff, K., Remmers, J., Poché, R., 2011. Bionomics of phlebotomine sand flies from three villages in Bihar, India. Journal of Vector Ecology 36 Suppl 1, S106–17. doi:10.1111/j.1948-7134.2011.00119.x.

[20] Shortt, H. E., 1945. Recent research on kala-azar in india. Transactions of the Royal Society of Tropical Medicine and Hygiene 39 (1), 13–31.

[21] Stauch, A., Duerr, H.-P., Picado, A., Ostyn, B., Sundar, S., Rijal, S., Boelaert, M., Dujardin, J.-C., Eichner, M., 2014. Model-based investigations of different vector-related intervention strategies to eliminate visceral leishmaniasis on the Indian subcontinent. PLoS Neglected Tropical Diseases 8 (4), e2810. doi:10.1371/journal.pntd.0002810.

[22] Stauch, A., Sarkar, R. R., Picado, A., Ostyn, B., Sundar, S., Rijal, S., Boelaert, M., Dujardin, J. C., Duerr, H. P., 2011. Visceral leishmaniasis in the indian subcontinent: Modelling epidemiology and control. PLoS Neglected Tropical Diseases 5 (11), 1–12. doi:10.1371/journal.pntd.0001405.

[23] Sundar, S., Sinha, P. K., Rai, M., Verma, D. K., Nawin, K., Alam, S., Chakravarty, J., Vaillant, M., Verma, N., Pandey, K., et al., 2011. Comparison of short-course multidrug treatment with standard therapy for visceral leishmaniasis in india: an open-label, non-inferiority, randomised controlled trial. The Lancet 377 (9764), 477–486.

[24] Tiwary, P., Kumar, D., Mishra, M., Singh, R. P., Rai, M., Sundar, S., 2013. Seasonal Variation in the Prevalence of Sand Flies Infected with Leishmania donovani. PLoS One 8 (4), e61370. doi:10.1371/journal.pone.0061370.
