# Supplementary material for: Elimination of visceral leishmaniasis in the Indian subcontinent: a comparison of predictions from three transmission models
Source: Epidemics. 2017 Mar;18:67–80. doi: 10.1016/j.epidem.2017.01.002 (PMC5340844; doi:10.1016/j.epidem.2017.01.002)
Supplement: Supplementary File S2 — Data description. [file mmc2.docx]

**Supplementary File 2: Further description of CARE data and parameter estimation**

Spatial, seasonal and annual variation in identified VL case numbers
The burdens of identified VL cases in 2012 in the 8 districts in the CARE dataset are given in Table S1. These have been calculated from the data by dividing the number of identified cases in each district by the total population of its *sub-districts* that had at least 1 case between January 2012 and June 2013. The sub-district populations are taken from the 2011 Census of India and population growth has been taken into account using the average yearly percentage population growth per district, derived from the change in the district populations between the 2001 and 2011 censuses (1). The identified VL case burden in 2012 varied significantly by district, ranging from 0.29 cases per 10,000 capita per year for West Champaran to 5.03/10,000/year for Saharsa, though for most districts it was between 1 and 2 cases per 10,000/year. Figure S1 shows the monthly numbers of onsets of VL symptoms from January 2012 to June 2013 for each of the 8 districts. There is clear seasonal variation in the numbers of cases in the five highest burden districts (Saharsa, East Champaran, Samastipur, Gopalganj and Khagaria), with an annual peak between January and April. This seasonal variation in case numbers has also been observed in other studies (2,3) (though from monthly numbers of diagnoses rather than onsets of symptoms), and is almost certainly driven by seasonal variation in sandfly abundance. The lag(s) between the peak(s) in the sandfly population (believed to be in June-August and October-November (4–6)) and that in the incidence may therefore hold valuable information about the duration of asymptomatic infection prior to clinical VL. In all districts but West Champaran, the numbers of cases decreased between January to June 2012 and January to June 2013.


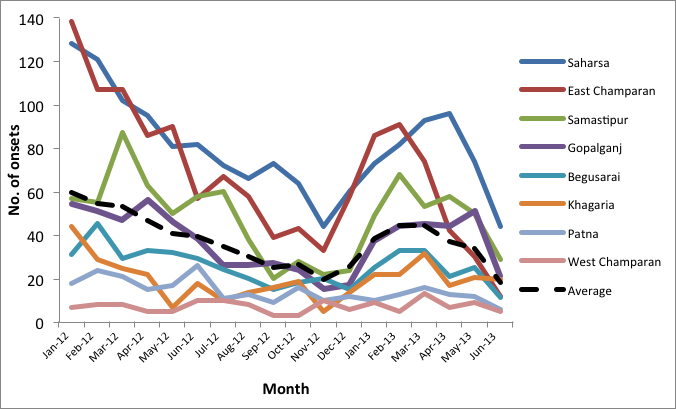


Figure S1 Monthly numbers of onsets of symptomatic VL cases for districts in CARE data. Dashed line shows average monthly number of cases across the districts.

| District | Number of identified VL cases ^*^ | | Population size^**^ | | Annual percentage growth rate (%) ^***^ | Burden of identified cases (cases per 10,000 population per year)^#^ | Mean duration onset of symptoms to treatment (OT) (days) | | Fraction T1 to T2^##^ | Level of IRS coverage(%) ^###^ | Start year of IRS |
| --- | --- | --- | --- | --- | --- | --- | --- | --- | --- | --- | --- |
|  | 2012  (Jan-Dec) | 2013 (Jan-June) | 2012 | 2013 | 2001-2011 | 2012 | 2012 | 2013 | 2012 | 2012 |  |
| Saharsa | 988 | 462 | 1963972 | 1998338 | 2.34 | 5.03 | 33.3 | 34.2 | 6.4% | 72.46 | 2011 |
| East Champaran | 884 | 335 | 5289197 | 5392537 | 2.61 | 1.67 | 53.5 | 44.1 | 7.3% | 63.16 | 2011 |
| Samastipur | 562 | 307 | 4401084 | 4476786 | 2.30 | 1.27 | 38.2 | 34.2 | 14.0% | 56.68 | 2011 |
| Gopalganj | 427 | 242 | 2625987 | 2660501 | 1.76 | 1.63 | 47.5 | 42.8 | 21.9% | 44.42 | 2011 |
| Begusarai | 311 | 148 | 3039108 | 3093054 | 2.37 | 1.02 | 21.6 | 22.5 | 10.8% | 62.09 | 2011 |
| Khagaria | 223 | 134 | 1730364 | 1764943 | 2.67 | 1.29 | 36.6 | 32.9 | 14.5% | 58.14 | 2011 |
| Patna | 192 | 70 | 5949098 | 6044879 | 2.15 | 0.32 | 44.9 | 43.0 | 9.4% | 58.70 | 2011 |
| West Champaran | 83 | 48 | 2879280 | 2935300 | 2.60 | 0.29 | 64.6 | 60.6 | 6.4% | 53.66 | 2011 |
| Total /  Average | 3670 | 1746 | 2787809 | 2836634 | 2.35 | 1.57 | 42.5 | 39.3 | 11.3% | 58.66 |  |

Table S1. Overview of CARE data and Census data (2001, 2011), input for geographical cross validation.

^*^ Numbers include 37 individuals with estimated onset dates between 1^st^ January 2012 and 30^th^ June 2013 from known diagnosis dates and mean district onset-to-diagnosis times

^**^ Estimated population size of affected sub-districts at 1^st^ of July 2012 and 1^st^ of April 2013.

^***^ District level population growth rates, calculated from the Indian census data 2001-2011 ^#^ Calculated as the total number of cases per district in 2012 divided by the population size of all affected sub-districts on the 1^st^ of July 2012 * 10,000

^##^ T1 = treatment 1 and T2= treatment 2

^###^ Calculated as the average of the percentage of patients houses that were sprayed in 2012 and the percentage of houses that were sprayed in the tola of patients in 2012

Official data on the annual numbers of VL cases in the 8 districts, collected by Thakur *et al* between 2006 and 2011, show a period of relatively constant numbers of cases between 2009 and 2011 (inclusive). This period of relative equilibrium is followed by a sharp decrease in incidence from 2011 to 2012 in the four districts with most cases (Saharsa, East Champaran, Samastipur and Gopalganj) (Figure S2). The districts with lower numbers of cases – Begusarai, Khagaria, Patna and West Champaran – also show a decrease in cases, but less steep. All 8 districts experienced a smaller decrease in the following period between 2012 and 2013. The numbers of cases in each district in 2012 recorded in the CARE data agree well with the official figures as presented by the State Surveillance Unit of the State Health Society Bihar (7) except for Samastipur, for which the number from the CARE data appears to be a considerable underestimate. Figure S3 shows the monthly numbers of VL diagnoses for all of Bihar from 2009 to 2012, which as in the CARE data have a clear seasonal pattern, with a peak between March and May (which is later than the peak in onsets due to the delay between onset and diagnosis). These data also show that the seasonal pattern in Bihar at state level was different for 2012, with a smaller peak in cases than in previous years followed by a large decrease in incidence over the rest of the year. This corresponds well with the general downward trend in cases at district level in 2012 observed in the CARE data, but suggests that the 2012 CARE data may not be representative of the `typical’ annual seasonal pattern.


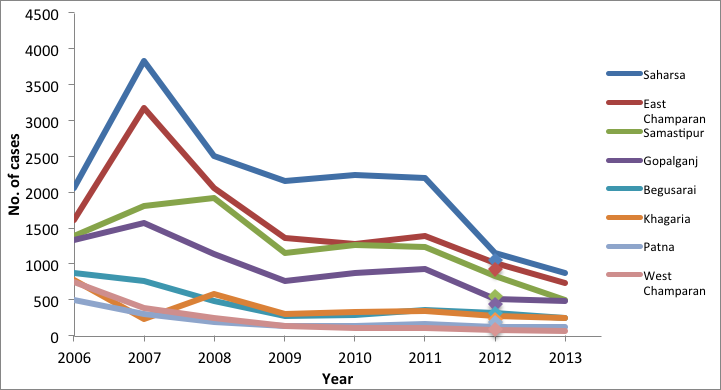


Figure S2. Yearly numbers of cases in 8 districts in Bihar from 2006 to 2011 from Thakur *et al* (9) in 2012 from (7) and in 2013 from (10). Dots represent the 2012 CARE data (onset of symptoms) which is for most districts lower compared to the data presented here by Thakur *et al*.

Data cleaning

The data were extensively cleaned to correct inconsistencies in dates reported by patients or their relatives (such as patients reporting being diagnosed before their onset of symptoms). Dates were corrected by crosschecking with all available information on durations of illness, dates of diagnosis and treatment for VL, and treatment prior to diagnosis for each case; and dates were only changed when there was sufficient information to be confident of the correct date. The self-reported dates of onset of symptoms and times between onset of


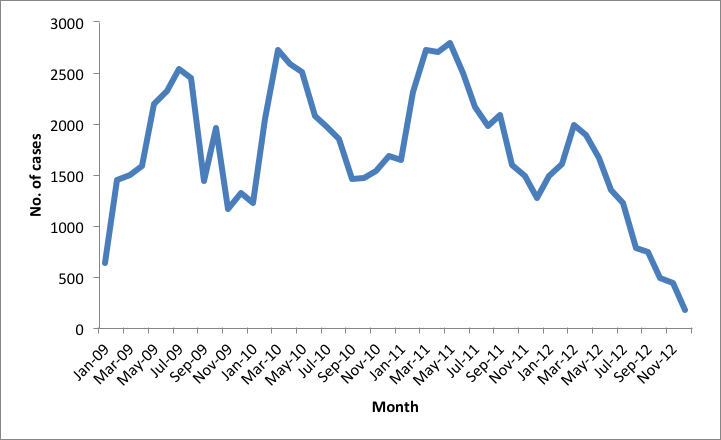


Figure S3. Monthly numbers of cases in CARE study districts from 2009-2013 from Annual Communicable Disease Surveillance Report, 2012, Bihar (7)

symptoms and start of treatment were used to fit the models. The monthly number of onsets per district was used as the incidence of ‘symptomatic untreated’ individuals in all models. Although the diagnosis dates are in theory more reliable (as more than half were taken from medical records), they include delays from onset to diagnosis, which differ between individuals and between districts. Hence the onset dates more accurately reflect, and enable fairer comparison of, seasonality in incidence across districts. Nevertheless, the diagnosis dates were used to correct inconsistencies in the onset dates.

There were 43 individuals in the data without an onset day or month, 42 of whom had a diagnosis date. The onset dates of the 42 individuals were estimated by subtracting the mean onset-to-diagnosis time for their district (for their year of diagnosis) from their diagnosis dates. Thirty-seven of the estimated onset dates fell between 1^st^ January 2012 and 30^th^ June 2013, and hence were included in the monthly numbers of onsets for each district (and in the figures in Table S1).

Onset-to-treatment time

Times between onset of symptoms and start of treatment varied significantly between districts with means ranging from 21.6 days in Begusarai to 64.6 days in West Champaran in 2012 (Table S1). The mean onset-to-treatment times decreased from 2012 to the first half of 2013 by 2 to 9 days for all districts apart from Saharsa and Begusarai, where the mean times both increased slightly. In both the Erasmus MC and Warwick models, this is accounted for by a step change in the mean duration of symptoms at the start of 2013.

Treatment duration

The majority of VL cases were treated with Miltefosine and the reported duration of 1^st^- and 2^nd^-line treatments was 28-30 days for most individuals, corresponding to taking a capsule daily or receiving daily injections for 4 weeks to a month. The average 1^st^ and 2^nd^ treatment durations are therefore taken as 28 days in all models.

Fraction of VL cases progressing to 2^nd^ treatment

Across all 8 districts, the average proportion of VL patients who received a 2^nd^ treatment was 11.3%. However, at district level the proportion ranged from 6.4% for Saharsa and West Champaran to 21.9% for Gopalganj. Hence we use the district-specific proportions in fitting the models.

PKDL
The data include some cases of PKDL, but the numbers per district are very low, so have not been used to fit the PKDL incidence in the Erasmus MC models (PKDL is not included in the Warwick model). Approximately 2.5% of all the VL cases in the data developed PKDL, which is comparable to estimates from other field studies (8). This value is therefore used to determine the flow between ‘putatively recovered’ to ‘PKDL’ in the Erasmus MC models, both for the fitting and the predictions.

IRS coverage

IRS coverage was calculated at district level from the CARE VL dataset and is based on the percentage coverage of spraying in 2012 in the house and/or neighborhood of the VL case. (Table S1) This approach is likely biased as only a subpopulation was interviewed in the CARE survey, however it represents the percentage coverage in VL endemic regions, which is what is being modeled in this study. Data on IRS coverage in the general population are unavailable for these years. For fitting the models, IRS at the calculated coverage levels was assumed to have started in 2011. When more detailed data become available in the near future, this can be included. A constant multiplication factor (representing the IRS efficacy), which was fitted, was used to convert the percentage coverage of IRS to the rate of reduction in sandfly density in the models.


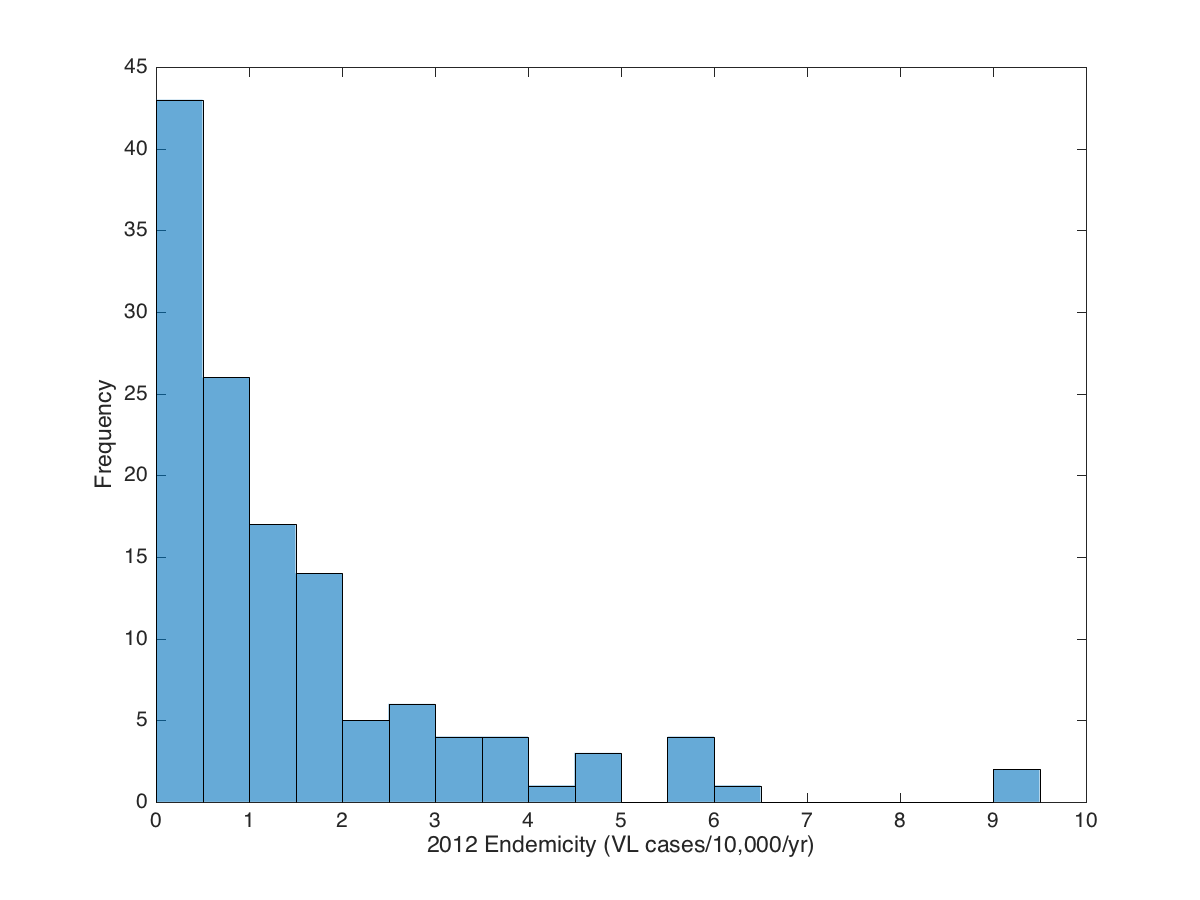


Figure S4. Distribution of 2012 sub-district endemicities for CARE districts

References

1. Ministry of Health and Family Welfare G of I. Family Welfare Statistics in India. 2011;306. Available from: http://www.2cnpop.net/uploads/1/0/2/1/10215849/mohfw_statistics_2011_revised_31_10_11.pdf

2. Malaviya P, Picado A, Singh SP, Hasker E, Singh RP, Boelaert M, et al. Visceral Leishmaniasis in Muzaffarpur District, Bihar, India from 1990 to 2008. Diemert DJ, editor. PLoS One [Internet]. 2011 Mar 4;6(3):e14751. Available from: http://dx.plos.org/10.1371/journal.pone.0014751

3. Burza S, Sinha PK, Mahajan R, Lima MA, Mitra G, Verma N, et al. Risk Factors for Visceral Leishmaniasis Relapse in Immunocompetent Patients following Treatment with 20 mg/kg Liposomal Amphotericin B (Ambisome) in Bihar, India. PLoS Negl Trop Dis. 2014;8(1):44.

4. Poché D, Garlapati R, Ingenloff K, Remmers J, Poché R. Bionomics of phlebotomine sand flies from three villages in Bihar, India. J Vector Ecol. 2011;36(SUPPL.1):106–17.

5. Picado A, Dash AP, Bhattacharya S, Boelaert M. Vector control interventions for visceral leishmaniasis elimination initiative in South Asia, 2005-2010. Indian J Med Res. India; 2012 Jul;136(1):22–31.

6. Malaviya P, Hasker E, Picado A, Mishra M, Van Geertruyden JP, Das ML, et al. Exposure to phlebotomus argentipes (diptera, psychodidae, phlebotominae) sand flies in rural areas of Bihar, India: The role of housing conditions. PLoS One. 2014;9(9):1–7.

7. State Surveillance Unit, State Health Society Bihar. Annual Communicable Disease Surveillance Report, 2012 Bihar. 2012.

8. Uranw S, Ostyn B, Rijal A, Devkota S, Khanal B, Menten J, et al. Post-kala-azar dermal leishmaniasis in nepal: A retrospective cohort study (2000-2010). PLoS Negl Trop Dis. 2011;5(12):1–7.

9. Thakur CP, Kumar A, Kumar A, Sinha K, Thakur S. A new method of kala-azar elimination : shifting the reservoir of infection from that village. Glob Adv Res J Med Med Sci. 2013;2(7):163–76.

10. Independent Commission on Development & Health in India. Elimination of Kala-Azar. 2014;
