# Supplementary material for: Elimination of visceral leishmaniasis in the Indian subcontinent: a comparison of predictions from three transmission models
Source: Epidemics. 2017 Mar;18:67–80. doi: 10.1016/j.epidem.2017.01.002 (PMC5340844; doi:10.1016/j.epidem.2017.01.002)
Supplement: Supplementary File S3 — Fitting procedure and Erasmus MC sensitivity analyses. [file mmc3.docx]

**Supplementary File 3: Geographical cross-validation of models, Erasmus MC sub-model selection, and sensitivity analyses**

**Cross-validation method**

We tested the models’ ability to reproduce censored data by means of a geographical cross-validation. The cross-validation was performed using a leave-one-out approach at the level of districts. Iteratively, the monthly number of VL onsets from January 2012 to June 2013 for one district were censored, and the model fitted to the data for the other 7 districts to estimate the IRS efficacy factor (Erasmus MC models, E0 and E1) or linear association between the district sandfly-to-human ratios (SHRs) and average 2012 case burdens from which the SHR for the censored district was estimated (Warwick model, W). The estimated IRS efficacy or SHR based on the 7 uncensored districts was used to estimate the monthly numbers of VL cases for the censored district. The mean onset-to-treatment times for 2012 and 2013, and 2012 IRS coverage level of the censored district were used in the simulation for the censored district. The ability of each model to reproduce the censored data was expressed in terms of the deviance of the model from the data summed over the eight iterations of censoring (Table S1). The model deviance is defined as twice the difference between the negative log-likelihood (LL) of the fitted model and the negative LL of a saturated model (i.e. one that fits the data exactly), as is presented for each model in Table S1. The lower the deviance, the closer the predictions of the model are to the data.

| Censored district | Model E0 | Model E1 | Model W |
| --- | --- | --- | --- |
| Saharsa | 60 | 95 | 39.1 |
| East Champaran | 89 | 88 | 1516.2 |
| Samastipur | 182 | 176 | 214.7 |
| Gopalganj | 195 | 176 | 263.3 |
| Begusarai | 181 | 239 | N/A |
| Khagaria | 67 | 78 | 332.0 |
| Patna | 84 | 100 | 69.6 |
| West Champaran | 31 | 36 | 1655.9 |
| Total deviance | 890 | 988 | 4090.8 |

Table S1. Model deviances for censored districts in geographical cross-validation. Values for models E0 and E1 are based on the selected sub-models with an early asymptomatic stage duration of 202 days, which were associated with the lowest deviances. Deviances of all other sub-models are presented in Table S3.

Although the geographical cross-validation enabled the predictive power of the models to be tested, fitting the models to the data from all 8 districts leads to better estimation of the fitted parameters. Therefore the models were also fitted to all districts without censoring, and these fitted parameter values were used for future predictions.

Models E0 and E1

*Estimation of IRS efficacy*

The geographical cross-validation was performed for each of the 18 sub-models (models E0 and E1 each with nine different assumptions about the duration of the early asymptomatic stage of VL). The district specific SHR was fitted to the Thakur data (1) for all 18 sub-models, to arrive at a pre-IRS equilibrium. Then the IRS efficacy parameter was fitted to the CARE data using the monthly numbers of VL onsets for the 7 uncensored districts via a maximum likelihood approach, assuming the data are Poisson-distributed. The estimated IRS efficacy was then used to predict the number of cases in the censored district for which the deviance per sub-model was calculated. Table S3 presents the deviances for all the 18 sub-models for the 8 iterations of censoring and also when fitting to all districts simultaneously. For both models E0 and E1, the sub-model with a duration of the early asymptomatic stage of 202 days best reproduced the censored data. These models were used for further simulations and forward predictions. Table S2 shows the deviances when all models are fitted to all of the CARE data.

**Table S2. Deviances of models E0, E1 and W when employing all available CARE.**

| District | Model E0 | Model E1 | Model W |
| --- | --- | --- | --- |
| Saharsa | 60.2 | 87.6 | 34.5 |
| East Champaran | 88.8 | 88.0 | 107.1 |
| Samastipur | 163.5 | 160.2 | 48.1 |
| Gopalganj | 183.2 | 163.8 | 32.3 |
| Beghusarai | 180.3 | 216.8 | 16.7 |
| Khagaria | 66.7 | 75.7 | 50.5 |
| Patna | 83.9 | 95.5 | 17.0 |
| West Champaran | 31.1 | 35.7 | 19.7 |
| Total deviance | 857.7 | 923.3 | 325.9 |

*Sensitivity analyses*

The deviances of the 18 sub-models in the sensitivity analyses of different average durations of immunity of one and five years are presented in Tables S4 and S5 respectively. The deviances of the 18 sub-models in the sensitivity analyses of different start year of IRS, 2010 and 2012, are presented in tables S6 and S7 respectively.

Model W
*Estimation of sandfly-to-human ratio*

The sandfly-to-human ratio in the censored district was estimated from the fitted SHRs in the other 7 districts by least squares linear regression of the average uncensored district identified case burdens in 2012 (see Table S1 in Supplementary File 2) against the fitted SHRs (Figure S1).

**Figure S1. Example of estimation of average sandfly-to-human ratio (SHR) for censored district using fitted SHRs and average identified VL case burdens in 2012 for 7 uncensored districts.** Censored district is East Champaran.

**Results**Figure 2 in the main text presents the estimated monthly numbers of cases in each censored district for the 3 models against the actual numbers from the data. Figure S2 in this document shows the estimated monthly numbers of cases for all districts employing all of the CARE data. For all models, the estimated numbers match the data relatively well across all the districts, although the models do not capture the extremities of the seasonal variation, in particular in the 4 districts with the highest number of cases (Saharsa, East Champaran, Samastipur and Gopalganj).

**Figure S2. Estimated monthly numbers of cases from the models when fitted to all districts without censoring.** Black dots show monthly numbers of cases from the CARE data.

Models E0 and E1 sensitivity analysis
*Duration of immunity*

When assuming a duration of immunity of one year, the models also fitted the data closely, but with a slightly higher overall deviance compared to the default duration of two years. When fitting the models with a duration of immunity of five years, extreme sandfly-to-human ratios were required to compensate for the corresponding relatively small population of susceptible individuals, and for the same reason a relatively long duration of the early asymptomatic stage (> 292 days) was required (Table S5) and only possible in model E0 which has a faster disease progression.

*Start year IRS*
In the sensitivity analysis for the start year of IRS control, both models E0 and E1 were able to predict the CARE data of the censored districts with IRS starting in January 2010 resulting in similar deviances compared to the default start year of 2011. Much lower IRS efficacy was needed to arrive at the CARE data due to the longer period of application of IRS between the pre-control equilibrium (fitted to Thakur *et al.*) and the start of the CARE data (Table S6). Fitting the models with a start year of IRS in January 2012, however, gave a much poorer fit to the CARE data (Table S7), and the maximum IRS efficacy of 100% was required in all the geographical cross-validations to reproduce the steep decrease in cases between 2012 and 2013 in the CARE data and between the Thakur and CARE data.

*Predictions*

The predictions by models E0 and E1 for the 9 durations of the early asymptomatic stage are presented in Figure S3. The difference in predictions of reaching the elimination target between the shortest (142 days) and longest (382 days) duration of early asymptomatic stage, ranges between 1 and 3 years, depending on the district, with the longest duration until elimination predicted by the model with the longest duration of early asymptomatic stage.

**Figure S3. Estimated VL incidence for all Erasmus MC sub-models when fitted to all districts without censoring.** The blue dot in 2010 represents the 2009-2010 average yearly incidence level in the data presented by Thakur *et al* (1), which was taken as the pre-control equilibrium incidence. The Thakur data consist of numbers of individuals that were treated for VL, which was linked to the ‘treatment 1’ stage in the model. The black dots show the monthly incidence by onset of symptoms from the CARE data. The black incidence lines overlay the lines of the best fitting sub-models, which is the sub-model with the early asymptomatic stage duration of 202 days in both models E0 and E1. The black dashed line represents the WHO elimination target.

| Model | Duration  early asymptomatic stage (days) | IRS efficacy (All districts) | **Deviance** | | | | | | | | | |
| --- | --- | --- | --- | --- | --- | --- | --- | --- | --- | --- | --- | --- |
|  |  |  | All districts | **Geographical cross-validation** | | | | | | | | |
|  |  |  |  | Without SAH | Without  ECH | Without  SAM | Without  GOP | Without  BEG | Without  KHA | Without  PAT | Without  WCH | Total  deviance |
| E0 | 382 | 1.000 | 1933 | 470 | 361 | 514 | 414 | 53 | 76 | 23 | 22 | 1933 |
|  | 352 | 1.000 | 1687 | 376 | 301 | 452 | 377 | 63 | 70 | 26 | 21 | 1688 |
|  | 322 | 1.000 | 1450 | 286 | 243 | 390 | 339 | 75 | 65 | 32 | 21 | 1450 |
|  | 292 | 1.000 | 1234 | 204 | 189 | 327 | 299 | 92 | 61 | 39 | 22 | 1233 |
|  | 262 | 1.000 | 1050 | 134 | 143 | 266 | 259 | 115 | 60 | 50 | 23 | 1050 |
|  | 232 | 1.000 | 916 | 84 | 107 | 210 | 219 | 144 | 61 | 64 | 26 | 916 |
|  | 202 | 0.999 | 858 | 60 | 89 | 182 | 195 | 181 | 67 | 84 | 31 | 890 |
|  | 172 | 0.948 | 878 | 69 | 83 | 178 | 191 | 212 | 69 | 99 | 35 | 936 |
|  | 142 | 0.905 | 1021 | 130 | 98 | 192 | 197 | 241 | 76 | 119 | 43 | 1096 |
| E1 | 382 | 1.000 | 1670 | 395 | 293 | 423 | 348 | 86 | 72 | 32 | 22 | 1670 |
|  | 352 | 1.000 | 1443 | 304 | 236 | 361 | 308 | 104 | 69 | 39 | 22 | 1443 |
|  | 322 | 1.000 | 1238 | 221 | 184 | 298 | 266 | 128 | 68 | 49 | 24 | 1238 |
|  | 292 | 1.000 | 1077 | 154 | 141 | 238 | 223 | 160 | 71 | 63 | 27 | 1077 |
|  | 262 | 1.000 | 988 | 114 | 115 | 199 | 191 | 203 | 79 | 83 | 33 | 1017 |
|  | 232 | 0.913 | 951 | 102 | 102 | 187 | 183 | 230 | 79 | 92 | 34 | 1010 |
|  | 202 | 0.829 | 923 | 95 | 88 | 176 | 176 | 239 | 78 | 100 | 36 | 988 |
|  | 172 | 0.749 | 928 | 104 | 76 | 168 | 171 | 254 | 79 | 112 | 40 | 1003 |
|  | 142 | 0.675 | 1030 | 162 | 76 | 171 | 173 | 285 | 86 | 134 | 47 | 1133 |

**Table S3**. **Deviances of Erasmus MC sub-models with start year of IRS in 2011 and duration of immunity of 2 years.** Deviances of the sub-models when fitted to all districts simultaneously (^A^) are presented in the first column ‘All districts’. Listed left of this column is the IRS efficacy based on the simultaneous fit to all districts. The deviances for the 8 censored districts are presented in the other columns, and are added together in the last column, which is listed as ‘Total deviance’, the deviance in this column that is closest to zero (shaded row) indicates the best-performing sub-model for models E0 and E1. The abbreviations used for the districts are as follows: SAH for Saharsa, ECH for East Champaran, SAM for Samastipur, GOP for Gopalganj, BEG for Begusarai, KHA for Khagaria, PAT for Patna and WCH for West Champaran.

**Table S4**. **Deviances of Erasmus MC sub-models with start year of IRS in 2011 and duration of immunity of 1 year.** Columns and abbreviations as in Table S3.

| Model | Duration  early asymptomatic stage (days) | IRS efficacy (All districts) | **Deviance** | | | | | | | | | |
| --- | --- | --- | --- | --- | --- | --- | --- | --- | --- | --- | --- | --- |
|  |  |  | All districts | **Geographical cross-validation** | | | | | | | | |
|  |  |  |  | Without SAH | Without  ECH | Without  SAM | Without  GOP | Without  BEG | Without  KHA | Without  PAT | Without  WCH | Total  deviance* |
| E0 | 382 | 1.000 | 2086 | 476 | 461 | 539 | 413 | 55 | 99 | 20 | 22 | 2086 |
|  | 352 | 1.000 | 1725 | 388 | 391 | 445 | 321 | 45 | 84 | 19 | 32 | 1725 |
|  | 322 | 1.000 | 1504 | 277 | 273 | 411 | 305 | 101 | 63 | 40 | 32 | 1504 |
|  | 292 | 1.000 | 1220 | 207 | 194 | 329 | 274 | 71 | 61 | 59 | 26 | 1220 |
|  | 262 | 1.000 | 1135 | 132 | 132 | 281 | 282 | 125 | 59 | 90 | 34 | 1135 |
|  | 232 | 1.000 | 877 | 83 | 107 | 219 | 229 | 107 | 64 | 46 | 23 | 877 |
|  | 202 | 0.997 | 884 | 62 | 90 | 206 | 196 | 182 | 70 | 95 | 24 | 924 |
|  | 172 | 0.944 | 890 | 66 | 82 | 192 | 199 | 195 | 69 | 108 | 35 | 946 |
|  | 142 | 0.897 | 985 | 108 | 96 | 211 | 201 | 231 | 70 | 91 | 38 | 1046 |
| E1 | 382 | 1.000 | 6151 | 462 | 157 | 915 | 770 | 1644 | 142 | 2024 | 37 | 6151 |
|  | 352 | 1.000 | 1655 | 313 | 322 | 132 | 169 | 210 | 70 | 76 | 362 | 1655 |
|  | 322 | 1.000 | 1970 | 258 | 211 | 101 | 117 | 325 | 69 | 35 | 857 | 1973 |
|  | 292 | 1.000 | 2123 | 143 | 182 | 228 | 254 | 35 | 94 | 1117 | 70 | 2124 |
|  | 262 | 1.000 | 2048 | 129 | 169 | 107 | 119 | 144 | 70 | 1287 | 55 | 2079 |
|  | 232 | 0.944 | 2166 | 104 | 103 | 269 | 277 | 340 | 90 | 1012 | 79 | 2274 |
|  | 202 | 0.849 | 947 | 86 | 88 | 213 | 225 | 232 | 79 | 49 | 43 | 1016 |
|  | 172 | 0.739 | 1087 | 108 | 105 | 283 | 114 | 421 | 112 | 24 | 37 | 1204 |
|  | 142 | 0.659 | 1185 | 133 | 73 | 177 | 173 | 310 | 111 | 73 | 221 | 1270 |

* Values of ‘Total deviance’ and deviance of ‘All districts’ are similar when the fitted IRS efficacy for both the cross-validation as well as the fitting to all districts arrives at a value of 1.0.

**Table S5**. **Deviances of Erasmus MC sub-models with start year of IRS in 2011 and duration of immunity of 5 years.** Columns and abbreviations as in Table S3.

| Model | Duration  early asymptomatic stage (days) | IRS efficacy (All districts) | **Deviance** | | | | | | | | | |
| --- | --- | --- | --- | --- | --- | --- | --- | --- | --- | --- | --- | --- |
|  |  |  | All districts | **Geographical cross-validation** | | | | | | | | |
|  |  |  |  | Without SAH | Without  ECH | Without  SAM | Without  GOP | Without  BEG | Without  KHA | Without  PAT | Without  WCH | Total  deviance |
| E0 | 382 | 1.000 | 21225 | 2727 | 3930 | 3964 | 2981 | 1242 | 1069 | 3357 | 1954 | 21225 |
|  | 352 | 1.000 | 21872 | 2662 | 3893 | 4055 | 3028 | 1354 | 1089 | 3743 | 2048 | 21872 |
|  | 322 | 1.000 | 21146 | 2506 | 3649 | 4024 | 3031 | 1300 | 1032 | 3632 | 1972 | 21146 |
|  | 292 | NA | NA | NA | NA | NA | NA | NA | NA | NA | NA | NA |
|  | 262 | NA | NA | NA | NA | NA | NA | NA | NA | NA | NA | NA |
|  | 232 | NA | NA | NA | NA | NA | NA | NA | NA | NA | NA | NA |
|  | 202 | NA | NA | NA | NA | NA | NA | NA | NA | NA | NA | NA |
|  | 172 | NA | NA | NA | NA | NA | NA | NA | NA | NA | NA | NA |
|  | 142 | NA | NA | NA | NA | NA | NA | NA | NA | NA | NA | NA |
| E1 | 382 | NA | NA | NA | NA | NA | NA | NA | NA | NA | NA | NA |
|  | 352 | NA | NA | NA | NA | NA | NA | NA | NA | NA | NA | NA |
|  | 322 | NA | NA | NA | NA | NA | NA | NA | NA | NA | NA | NA |
|  | 292 | NA | NA | NA | NA | NA | NA | NA | NA | NA | NA | NA |
|  | 262 | NA | NA | NA | NA | NA | NA | NA | NA | NA | NA | NA |
|  | 232 | NA | NA | NA | NA | NA | NA | NA | NA | NA | NA | NA |
|  | 202 | NA | NA | NA | NA | NA | NA | NA | NA | NA | NA | NA |
|  | 172 | NA | NA | NA | NA | NA | NA | NA | NA | NA | NA | NA |
|  | 142 | NA | NA | NA | NA | NA | NA | NA | NA | NA | NA | NA |

**Table S6**. **Deviances of Erasmus MC sub-models with start year of IRS in 2010 and duration of immunity of 2 years.** Columns and abbreviations as in Table S3.

| Model | Duration  IHP (days) | IRS efficacy (All districts) | **Deviance** | | | | | | | | | |
| --- | --- | --- | --- | --- | --- | --- | --- | --- | --- | --- | --- | --- |
|  |  |  | All districts | **Geographical cross-validation** | | | | | | | | |
|  |  |  |  | Without SAH | Without  ECH | Without  SAM | Without  GOP | Without  BEG | Without  KHA | Without  PAT | Without  WCH | Total  deviance |
| E0 | 382 | 1.000 | 904 | 54 | 159 | 178 | 186 | 175 | 73 | 81 | 26 | 933 |
|  | 352 | 0.959 | 890 | 51 | 157 | 171 | 181 | 195 | 75 | 88 | 27 | 944 |
|  | 322 | 0.916 | 875 | 49 | 154 | 165 | 175 | 197 | 74 | 91 | 27 | 931 |
|  | 292 | 0.875 | 861 | 50 | 150 | 158 | 169 | 199 | 73 | 95 | 28 | 921 |
|  | 262 | 0.839 | 852 | 55 | 146 | 151 | 163 | 202 | 72 | 100 | 30 | 917 |
|  | 232 | 0.806 | 855 | 70 | 142 | 145 | 157 | 207 | 71 | 106 | 32 | 930 |
|  | 202 | 0.779 | 887 | 103 | 143 | 143 | 153 | 216 | 72 | 115 | 35 | 979 |
|  | 172 | 0.760 | 988 | 173 | 155 | 150 | 154 | 232 | 76 | 130 | 41 | 1111 |
|  | 142 | 0.753 | 1246 | 332 | 199 | 178 | 168 | 264 | 88 | 156 | 52 | 1435 |
| E1 | 382 | 0.878 | 940 | 65 | 155 | 165 | 169 | 240 | 82 | 96 | 29 | 1002 |
|  | 352 | 0.821 | 929 | 64 | 151 | 160 | 165 | 243 | 81 | 98 | 29 | 993 |
|  | 322 | 0.766 | 916 | 64 | 146 | 154 | 161 | 247 | 80 | 101 | 30 | 983 |
|  | 292 | 0.712 | 903 | 65 | 140 | 146 | 156 | 252 | 80 | 105 | 31 | 975 |
|  | 262 | 0.661 | 892 | 71 | 132 | 138 | 150 | 258 | 79 | 110 | 32 | 972 |
|  | 232 | 0.612 | 890 | 87 | 124 | 130 | 144 | 268 | 79 | 117 | 34 | 983 |
|  | 202 | 0.566 | 914 | 123 | 117 | 123 | 139 | 283 | 80 | 128 | 38 | 1030 |
|  | 172 | 0.525 | 1004 | 211 | 119 | 119 | 135 | 309 | 85 | 147 | 44 | 1168 |
|  | 142 | 0.491 | 1272 | 438 | 148 | 129 | 139 | 359 | 101 | 180 | 56 | 1552 |

**Table S7**. **Deviances of Erasmus MC sub-models with start year of IRS in 2012 and duration of immunity of 2 years.** Columns and abbreviations as in Table S3.

| Model | Duration  IHP (days) | IRS efficacy (All districts) | **Deviance** | | | | | | | | | |
| --- | --- | --- | --- | --- | --- | --- | --- | --- | --- | --- | --- | --- |
|  |  |  | All districts | **Geographical cross-validation** | | | | | | | | |
|  |  |  |  | Without SAH | Without  ECH | Without  SAM | Without  GOP | Without  BEG | Without  KHA | Without  PAT | Without  WCH | Total  deviance |
| E0 | 382 | 1.000 | 5729 | 2022 | 1223 | 1303 | 849 | 46 | 202 | 32 | 52 | 5729 |
|  | 352 | 1.000 | 5524 | 1949 | 1169 | 1263 | 828 | 42 | 194 | 29 | 50 | 5524 |
|  | 322 | 1.000 | 5293 | 1865 | 1108 | 1218 | 804 | 38 | 185 | 26 | 48 | 5293 |
|  | 292 | 1.000 | 5037 | 1772 | 1039 | 1168 | 778 | 34 | 176 | 23 | 46 | 5037 |
|  | 262 | 1.000 | 4751 | 1669 | 962 | 1112 | 748 | 31 | 165 | 20 | 44 | 4751 |
|  | 232 | 1.000 | 4433 | 1552 | 874 | 1049 | 714 | 30 | 154 | 18 | 42 | 4433 |
|  | 202 | 1.000 | 4085 | 1424 | 775 | 979 | 675 | 31 | 142 | 17 | 41 | 4085 |
|  | 172 | 1.000 | 3713 | 1286 | 665 | 901 | 633 | 38 | 130 | 20 | 41 | 3713 |
|  | 142 | 1.000 | 3349 | 1144 | 548 | 821 | 589 | 57 | 120 | 28 | 43 | 3349 |
| E1 | 382 | 1.000 | 5506 | 1993 | 1168 | 1236 | 811 | 37 | 190 | 26 | 46 | 5506 |
|  | 352 | 1.000 | 5303 | 1918 | 1114 | 1197 | 790 | 34 | 183 | 23 | 44 | 5303 |
|  | 322 | 1.000 | 5074 | 1833 | 1053 | 1152 | 765 | 33 | 175 | 21 | 43 | 5074 |
|  | 292 | 1.000 | 4818 | 1738 | 984 | 1102 | 737 | 32 | 166 | 18 | 41 | 4818 |
|  | 262 | 1.000 | 4532 | 1633 | 905 | 1045 | 705 | 32 | 157 | 16 | 39 | 4532 |
|  | 232 | 1.000 | 4217 | 1516 | 816 | 980 | 667 | 35 | 148 | 16 | 38 | 4217 |
|  | 202 | 1.000 | 3879 | 1392 | 717 | 908 | 624 | 43 | 139 | 18 | 38 | 3879 |
|  | 172 | 1.000 | 3537 | 1265 | 610 | 830 | 576 | 61 | 132 | 24 | 40 | 3537 |
|  | 142 | 1.000 | 3250 | 1153 | 505 | 752 | 525 | 96 | 132 | 41 | 47 | 3250 |

**References**

1. Thakur CP, Kumar A, Kumar A, Sinha K, Thakur S. A new method of kala-azar elimination : shifting the reservoir of infection from that village. Glob Adv Res J Med Med Sci. 2013;2(7):163–76.
