# Supplementary material for: Elimination of visceral leishmaniasis in the Indian subcontinent: a comparison of predictions from three transmission models
Source: Epidemics. 2017 Mar;18:67–80. doi: 10.1016/j.epidem.2017.01.002 (PMC5340844; doi:10.1016/j.epidem.2017.01.002)
Supplement: Supplementary File S4 — Description to Supplementary File 5. [file mmc4.docx]

**Supplementary File 4: Age-structured system of ordinary differential equations for visceral leishmaniasis transmission by Erasmus MC**

R-package ‘VLode’ version 0.2.0

Download the Supplementary File 5.tgz file.

install.packages(devtools)

library(devtools)

setwd([“*path where ‘Supplementary File 5.tgz’ -file is located”*])

install("VLode")

library(VLode)

?VLode for examples and execution
