# Supplementary material for: Elimination of visceral leishmaniasis in the Indian subcontinent: a comparison of predictions from three transmission models
Source: Epidemics. 2017 Mar;18:67–80. doi: 10.1016/j.epidem.2017.01.002 (PMC5340844; doi:10.1016/j.epidem.2017.01.002)
Supplement: Supplementary File S5 — R-package ‘VLode’ of age-structured system of ordinary differential equations for visceral leishmaniasis transmission developed by Erasmus MC. [file mmc5.zip › VLode/html/00Index.html]

R: Age-structured system of ordinary differential equations for VL
transmission

# Age-structured system of ordinary differential equations for VL transmission

---

## Documentation for package ‘VLode’ version 0.2.0

- DESCRIPTION file.

## Help Pages

|  |  |
| --- | --- |
| age\_ode\_cpp | Update an age-structured derivative (vector) for aging. |
| aggr\_age | Aggregate range of positions in a vector. |
| IRS\_fun | Function for switching on IRS. |
| season\_fun | Function for seasonal sandfly abundance. |
| update\_parameters | Function to update conditional parameters |
| VLode | Function to integrate system of ordinary differential equations. |
